# Using carpet plots to analyze blood transit times in the brain during hypercapnic challenge magnetic resonance imaging

**DOI:** 10.3389/fphys.2023.1134804

**Published:** 2023-02-15

**Authors:** Bradley Fitzgerald, Jinxia Fiona Yao, Lia M. Hocke, Blaise deB. Frederick, Christiaan Hendrik Bas van Niftrik, Yunjie Tong

**Affiliations:** ^1^ Elmore Family School of Electrical and Computer Engineering, Purdue University, West Lafayette, IN, United States; ^2^ Weldon School of Biomedical Engineering, Purdue University, West Lafayette, IN, United States; ^3^ McLean Imaging Center, McLean Hospital, Belmont, MA, United States; ^4^ Department of Psychiatry, Harvard Medical School, Boston, MA, , United States; ^5^ Department of Neurosurgery, University Hospital Zurich, Zurich, Switzerland

**Keywords:** carpet plot, hypercapnia fMRI, cerebral transit time, dynamic susceptibility contrast imaging, structural similarity index

## Abstract

Blood arrival time and blood transit time are useful metrics in characterizing hemodynamic behaviors in the brain. Functional magnetic resonance imaging in combination with a hypercapnic challenge has been proposed as a non-invasive imaging tool to determine blood arrival time and replace dynamic susceptibility contrast (DSC) magnetic resonance imaging, a current gold-standard imaging tool with the downsides of invasiveness and limited repeatability. Using a hypercapnic challenge, blood arrival times can be computed by cross-correlating the administered CO_2_ signal with the fMRI signal, which increases during elevated CO_2_ due to vasodilation. However, whole-brain transit times derived from this method can be significantly longer than the known cerebral transit time for healthy subjects (nearing 20 s vs. the expected 5–6 s). To address this unrealistic measurement, we here propose a novel carpet plot-based method to compute improved blood transit times derived from hypercapnic blood oxygen level dependent fMRI, demonstrating that the method reduces estimated blood transit times to an average of 5.32 s. We also investigate the use of hypercapnic fMRI with cross-correlation to compute the venous blood arrival times in healthy subjects and compare the computed delay maps with DSC-MRI time to peak maps using the structural similarity index measure (SSIM). The strongest delay differences between the two methods, indicated by low structural similarity index measure, were found in areas of deep white matter and the periventricular region. SSIM measures throughout the remainder of the brain reflected a similar arrival sequence derived from the two methods despite the exaggerated spread of voxel delays computed using CO_2_ fMRI.

## 1 Introduction

Measurements related to blood arrival time throughout the brain represent important hemodynamic metrics in several contexts. Arrival time measurements are useful in characterizing cerebral hemodynamic behavior in patients; examples include the demonstration of prolonged blood arrival time in patients with multiple sclerosis (Paling et al., 2014), internal carotid artery occlusion (Bokkers et al., 2008), Moyamoya (Donahue et al., 2016), and stroke (Chalela et al., 2000; Macintosh et al., 2010). Additionally, computation of accurate blood arrival times is a key step in computing cerebral vascular reactivity (Niftrik et al., 2017; Yao et al., 2021) and is an important factor to consider when analyzing collateral perfusion (Zaharchuk et al., 2011). Further, blood transit time (the time taken for blood to pass through a region or whole of the brain) can be derived from arrival time measurements. Transit time can serve as a benchmark for evaluating the quality of region-specific (or voxel-wise) arrival time measurements, as the metric can be easily compared with known whole-brain blood transit times. Thus, the development of reliable, safe, and repeatable techniques for measuring cerebral blood arrival times and the associated cerebral blood transit times is important for the continued development of our understanding of blood dynamics in the brain.

Dynamic susceptibility contrast (DSC) imaging is frequently used to study magnetic resonance imaging (MRI) brain perfusion by imaging the first pass of an intravenously injected gadolinium-based contrast agent through the brain (Lentschig et al., 1998). DSC-MRI is a reliable reference standard for blood flow-related measurements, including transit time. However, it is a an invasive imaging technique and the side-effects of gadolinium-based agents remaining in the human body are still debatable (Essig et al., 2013), highlighting the need for a safer and more convenient alternative. Several different alternative methods for measurement of hemodynamic metrics, based on blood oxygen level dependent (BOLD) MRI, have been proposed (Aso et al., 2020; Bhogal et al., 2022; Sayin et al., 2022). Recently, an increasing number of studies have used elevated CO_2_ levels as a regressor to estimate the CO_2_/blood arrival time *via* BOLD MRI (Blockley et al., 2011; Thomas et al., 2013a; Duffin et al., 2015; Donahue et al., 2016). It is known that CO_2_ is a vasodilator, meaning that elevated CO_2_ arriving to a region of the brain can cause an increase in regional blood flow and volume, resulting in increased BOLD signals. Thus, blood flow can be observed by tracking the passage of the CO_2_ throughout the brain. BOLD-CO_2_ MRI offers advantages over DSC-MRI as a blood-tracking method, since CO_2_-MRI is a non-invasive technique and there are no adverse side-effects of inhaling elevated CO_2_ within a suitable range.

For CO_2_-MRI, a voxel’s estimated CO_2_/blood arrival time is commonly represented by the time delay corresponding to maximum cross-correlation coefficient (MCCC) between the voxel’s BOLD signal and the partial pressure of end-tidal CO_2_ concentration (P_ET_CO_2_) measurement (Blockley et al., 2011; Poublanc et al., 2013; Niftrik et al., 2017). However, this delay time often overestimates the true CO_2_ arrival time, leading to overestimation in whole-brain blood transit time assessment. This overestimation is a result of the brain’s varying hemodynamic response to CO_2_, as different brain regions differ in the time taken for local tissues to respond the arrival of the increased CO_2_, leading to various shapes of the BOLD signal waveform deformations from the P_ET_CO_2_ measurement (Duffin et al., 2015; Golestani et al., 2015; Poublanc et al., 2015; Prokopiou et al., 2019). Hence the cross-correlation time delay from the deformed BOLD signal does not purely reflect the signal onset (i.e., CO_2_ arrival time), but is also influenced by the CO_2_ hemodynamic response of the brain region. Simulations from a previous study showed that performing cross-correlation of the measured CO_2_ with various shapes of the BOLD signal waveform with a shared breakpoint (representing the same moment of CO_2_ arrivals) from the baseline can obtain different delay time values when the true delay should be equivalent (Yao et al., 2021). Depending on the extent of the distortion of the BOLD waveform from the CO_2_ measurement, one can obtain a delay time offset as large as 20 s (Yao et al., 2021). One study of 25 healthy subjects reported that the estimated arterial CO_2_ arrival times derived from the maximum cross-correlation method can have an average span of 20.1 s across the whole brain, with a span of 15.9 s for gray matter (GM) and 25.5 s for white matter (WM) (Niftrik et al., 2017), which is inconsistent with the fact that the whole-brain blood transit time is approximately 5–6 s on average (Hoffmann et al., 2000).

Thus, it is important to evaluate the effects of these signal deformations on the accuracy of the CO_2_-derived delay maps. This evaluation can be discussed through two different perspectives: first, the accuracy of the specific voxel-wise CO_2_-derived delay time values can be evaluated. Second, ordering of the voxels throughout the brain based on CO_2_-derived delay time values suggests a sequence of voxels corresponding to the arrival paths of the CO_2_ “bolus”. This sequence of voxels can also be evaluated for accuracy separately from evaluation of the delay values themselves. The use of a carpet plot to analyze CO_2_-MRI data holds potential to assist with better understanding this issue of widely spread CO_2_-derived delay times computed *via* the MCCC method.

A carpet plot is a 2-dimensional voxel vs. time matrix showing BOLD signal intensities, which was initially used within MRI-related studies for assessing quality of MRI signals (especially for the detection of motion artifacts) (Power, 2017). We previously made use of carpet plots for calculating the time taken for blood to pass through the brain (i.e., transit time) by ordering voxels according to delays (based on the low frequency oscillation signal delay time relative to the global averaged signal) computed using resting-state functional MRI (rs-fMRI) signals (Fitzgerald et al., 2021). The derived blood transit time from reconstructed resting-state carpet plots was shown to be comparable with that from DSC-MRI carpet plots. The same methodology can be applied to CO_2_-MRI data to create CO_2_-based carpet plots, in which observable signal patterns could prove useful in assessing the impact of distorted BOLD signals (caused by variations in reactivity to CO_2_ throughout the brain) on the assigned venous blood/CO_2_ arrival times, as well as in computing brain blood transit times using CO_2_-MRI data.

In this follow-up study, we apply this novel methodology to construct sorted carpet plots from CO_2_-challenge BOLD fMRI data. The purpose of this study is twofold: first, we use observed patterns in these carpet plots to group voxels based on their assigned CO_2_-derived delay times and demonstrate improved estimates of the blood transit time (through the majority of the brain) which are closer to the expected transit time values than those implied by the wide distribution of delay times computed from the cross-correlation method. Second, we aim to provide an analysis of the similarity between voxel-wise delay maps derived from CO_2_-MRI and from DSC-MRI. To this end, we use the carpet plot voxel groupings to examine how the delay times computed from the two methods compare using the “structure” element of the structural similarity index measure (SSIM) (Zhou et al., 2004). In addition, we examine patterns in the BOLD signal of voxels within these groups to move towards an understanding of how varying reactivity affects the sequential ordering of assigned CO_2_ delay times throughout the brain.

We acknowledge that conditions of brain perfusion differ between DSC-MRI and CO_2_-MRI; here we simply aim to investigate whether similar perfusion information (i.e., blood transit time) can be derived from CO_2_-MRI by utilizing our novel carpet plot methodology to tease out the confounding effects induced by the CO_2_ challenge. Such analysis helps to evaluate the possibility of using CO_2_-MRI as an alternative to DSC-MRI in the measurement of the blood transit time.

## 2 Materials and methods

### 2.1 Data acquisition

The Institutional Review Boards (IRB) of the institutions at which datasets were collected (McLean Hospital for DSC-MRI, Purdue University for CO_2_ challenge MRI) approved all experimental protocols used in this study. All experiments followed the ethical principles of the Belmont Report, and all subjects provided written informed consent. DSC-MRI data from eight healthy subjects (1F, 7M, mean ± s.d., 33 ± 12 years) were acquired using a Siemens TIM Trio 3T scanner (Siemens Medical Solutions, Malvern, PA) with 32-channel phased array head matrix coil. A gadolinium contrast agent was given by intravenous injection for the DSC-MRI scans (TR/TE = 1510/21 m, voxel size = 1.8 × 1.8 × 3.5 mm^3^, duration = 180 s). Detailed acquisition information for DSC-MRI can be found in a previous publication by Tong et al. (2017).

CO_2_ challenge data was collected from a separate set of eleven subjects (5F, 6M, age ±s.d., 22.7 ± 4.4 years; three female subjects were excluded due to dropouts and/or poor quality of data) using a 3T GE Discovery MR750 MR scanner. Two out of the eight remaining participants (Subjects 1 and 2, scanned during the protocol testing phase before parameters were adjusted for the remaining subjects) underwent fMRI scanning with the following parameters: (TR/TE = 800/30 ms, voxel size = 3.75 × 3.75 × 2.5 mm^3^, duration = 600 s). The remaining participants (Subjects 3–8) were scanned with the following parameters: (TR/TE = 1000/30 ms, voxel size = 3 × 3 × 3 mm^3^, duration = 600 s). The elevated CO_2_ challenge was controlled by a programmable computer-based gas delivery system (RespirAct, Thornhill Research Inc., Toronto, Canada) (Fisher, 2016). Each subject was fitted with a plastic face mask (covering the nose and the mouth) connected with a breathing circuit before entering the MRI scanner room. The breathing protocol consisted of 2 minutes of the “baseline” CO_2_ level (i.e., the resting CO_2_ level), followed by 2 minutes of elevated (10 mmHg higher than baseline) CO_2_, 2 minutes of baseline CO_2_, 2 minutes of elevated CO_2_, and two final minutes of baseline CO_2_. The system controlled and recorded the end-tidal partial pressure of CO_2_ (P_ET_CO_2_) time series.

### 2.2 Data preprocessing

Data were preprocessed using the FMRIB Software Library (FSL, Oxford University, United Kingdom, v5.0 for DSC-MRI data, v6.0 for the CO_2_-MRI data, https://fsl.fmrib.ox.ac.uk/fsl/fslwiki) (Jenkinson et al., 2012). Preprocessing included motion correction, slice-time correction, brain extraction, and spatial smoothing (3 mm for DSC-MRI scans, 5 mm for CO_2_-MRI scan).

### 2.3 Constructing DSC delay maps

**FIGURE 1:**
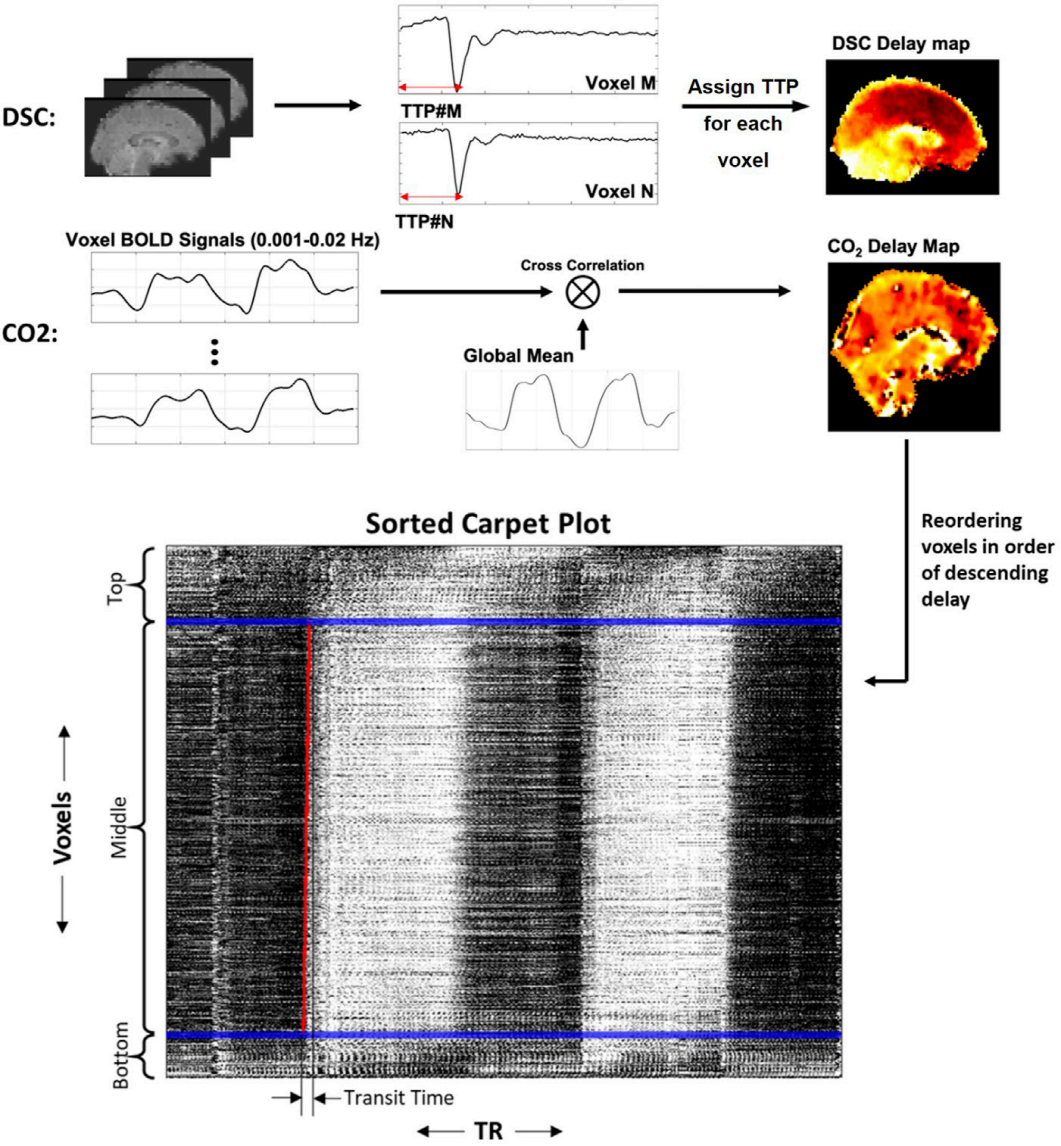
Methods for creating DSC delay maps, CO_2_ delay maps and carpet plots. DSC delay maps were created by computing the voxel-wise time to peak (TTP) metric, representing the delay until peak signal decrease was obtained, which corresponds to the arrival of the gadolinium bolus in a given voxel. CO_2_ delay maps were computed by performing voxel-wise cross-correlation between individual voxel time series and the global averaged time series and selecting the delay value which resulted in maximum absolute correlation. Voxels were then sorted in descending order according to the computed delay values, and the 2D carpet plot image was created by displaying the time series of all brain voxels in this sorted order (top representing largest delay, bottom representing smallest delay).

Gadolinium bolus delay maps were created for each DSC-MRI subject, where delay values represent the time-of-arrival of the gadolinium bolus in each brain voxel (see Figure 1). This voxel-specific delay is represented by the time to peak (TTP), which is computed as the time between initial injection until the maximum dip of the DSC signal loss. TTP was chosen over T0 (interval between injection and its first detection) because the peak can be easily and more accurately identified than the detection of the first arrival. TTP was calculated by the program Perfx using a gamma function fitting with temporal interpolation (developed by Chris Rorden, www.mccauslandcenter.sc.edu/CRNL/tools/pwi). After interpolation, the temporal resolution of DSC-MRI data for TTP was 0.0001 s.

### 2.4 Constructing CO_2_ delay maps

CO_2_ delay maps were computed using MATLAB (version R2017b or later, www.mathworks.com/). The brain-masked fMRI data was then arranged into a 2D matrix, where each row contains the measured BOLD time series for a voxel. Each voxel time series was detrended (MATLAB *detrend*) and normalized by dividing by the standard deviation of the time series. The global average time series was computed, detrended and normalized in the same way. For each voxel, the time series was oversampled by a factor of 10 (MATLAB *interpft*) to improve the temporal resolution. For the CO_2_ delay map calculation, the time series was filtered using a fourth-order Butterworth bandpass filter with bandwidth 0.001–0.02 Hz to extract the very low frequency oscillations associated with CO_2_-dependent fluctuations in the BOLD signal. The filtered time series was then compared with the global average time series (also oversampled by the same factor and filtered into the same frequency band) *via* cross-correlation, and the shifting-index which produced the maximum absolute correlation value between the two signals was recorded as the “delay” value for that voxel. We note that the use of the maximum absolute correlation allowed some voxel delays to be associated with a negative correlation, which is discussed in more detail in the Discussion section. Full-brain CO_2_ delay maps for each subject were computed using this method. These delay maps were then registered to a standard template space (Fonov et al., 2009), temporally aligned by subtracting the mean delay values, and averaged to produce a subject-averaged CO_2_ delay map.

### 2.5 Constructing CO_2_ carpet plots

**FIGURE 2:**
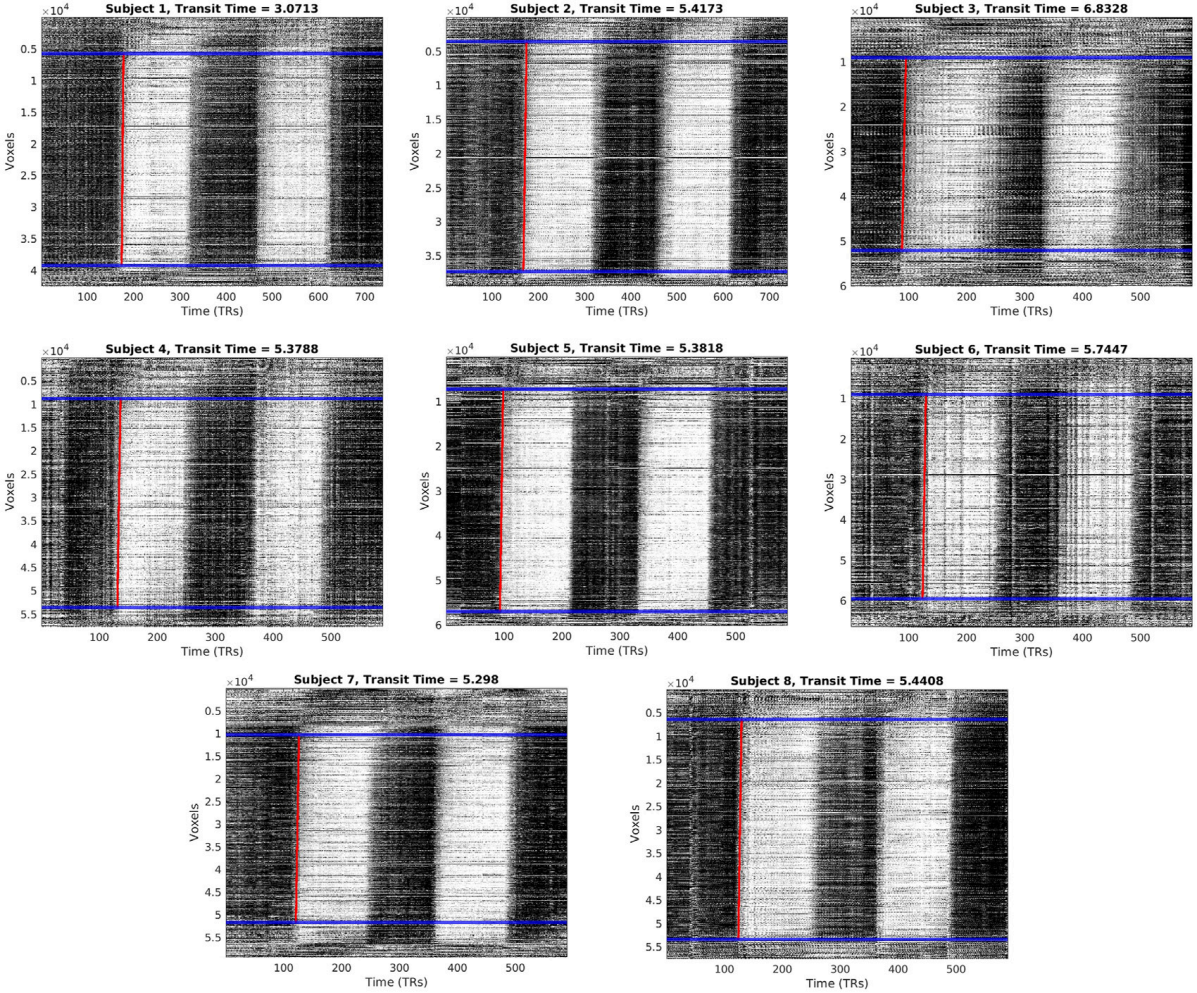
Carpet plots and transit times for each CO_2_ subject. Blue lines indicate boundaries for image cropping before edge detection. Red lines indicate the computed edge associated with the arrival of the CO_2_ bolus.

The rows of the fMRI data matrix (where rows are voxels and columns are time points) were reordered based on descending delay values (see Figure 1; longest delay voxels at top of image, shortest delay values at bottom of image), creating a sorted carpet plot. Visual inspection of this carpet plots demonstrates that most voxel time series in the center of the image form a linear edge, while a portion of voxel time series at the top and bottom of the image (representing voxels with comparatively high or low delays, respectively) do not follow this linear edge trend. This observation is relevant because it has been shown that carpet plots constructed from DSC-MRI data yield a near-linear blood-arrival edge covering nearly all voxels (above 95%) in the brain with reasonable associated transit times (Fitzgerald et al., 2021). Using DSC-MRI as a reference standard, this suggests that isolating voxels in the CO_2_ carpet plot which follow this linear trend may be useful in computing better estimates of the blood transit time. To this end, each carpet plot was divided into three vertical sections. These sections were created by defining the middle section such that it contained all voxels with delays inside a 20-s window centered at the median delay time (see window edges defined by blue lines in Figures 1, 2). The width of this window was chosen based on empirical results with visual confirmation, which suggested that such a window size served as a conservative but reliable method that could be applied consistently across all subjects to isolate those voxels which followed the linear edge trend. A brief further discussion of this window, the effect of varying its width, and testing of a subject-specific adaptive window width is presented in the Supplementary Section S1.

### 2.6 Carpet plot analysis

A slope detection program, introduced and discussed in our previous study (Fitzgerald et al., 2021), was used to detect and fit a linear line to the first CO_2_-arrival edge present in the middle section of the CO_2_ carpet plot, illustrated as a red line in Figure 1. Details regarding this slope detection program can be found in the previous publication (Fitzgerald et al., 2021); to summarize, the program estimates the linear edge present by fitting a linear trend line to a set of data points, where the data points represent the horizontal (time) point of maximum increase (i.e., maximum derivative). As such, the detected linear edge reflects the time of maximum CO_2_-induced BOLD signal increase. One adjustment to the described program was made: voxel time series within the sorted carpet plot were first frequency filtered (0.001–0.02 Hz) in order to capture the signal variation resulting from CO_2_ arrival while removing the variation resulting from low frequency oscillations (0.01–0.1 Hz) present in rs-fMRI data. After computation, a nearly vertical edge that is tilted slightly to the right can be observed due to the flow over time of the CO_2_-arrival-based BOLD signal rise throughout the brain. The horizontal (time) duration of this estimated edge line was recorded as the transit time (i.e., time taken for CO_2_ bolus to traverse all voxels present in the cropped carpet plot). Only transit times for the first carpet plot edge, corresponding to the first CO_2_ bolus given, is reported here. This choice was made in order to avoid unknown complications arising from any residual effects of the first CO_2_ bolus which might affect blood flow behavior during the second CO_2_ bolus (see Supplementary Figure S2 for transit times of the second edge).

**FIGURE 3:**
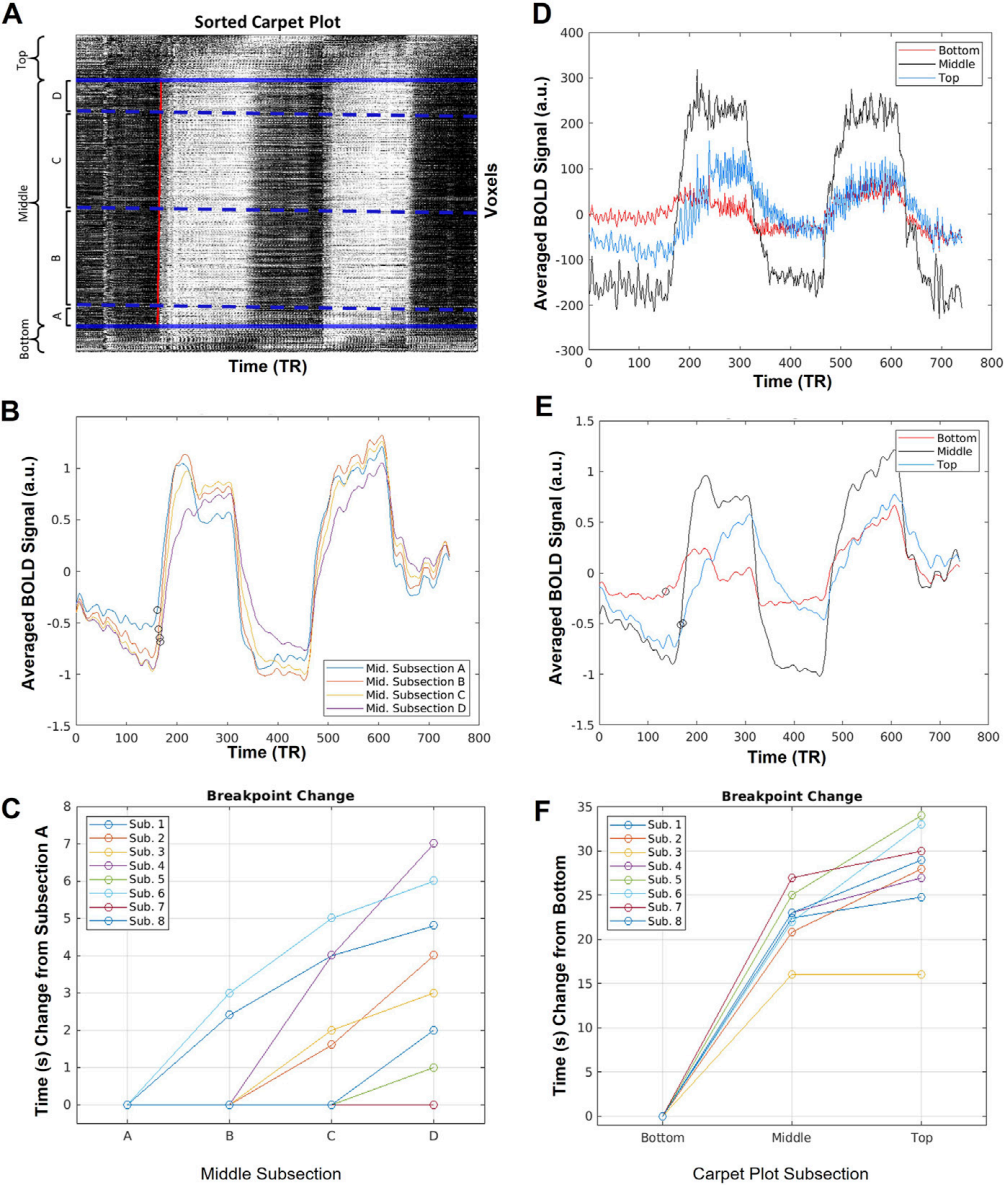
Sample averaged time series computed during carpet plot analysis. **(A)** Display of example sorted carpet plot, where the red line represents the edge computed during edge detection applied to the cropped carpet plot. Solid blue lines indicate the boundary of regions removed before edge detection, and divide the carpet plot into the “top”, “middle”, and “bottom” regions, as labeled. Dotted blue lines indicate boundaries for four subsections, (A–D), of the middle region; these subsections are defined by grouping voxels with assigned delay times falling within four 5-s windows (a division, into four groups, of the 20-s span of delays assigned to voxels in the middle section). **(B)** Display of the averaged time series of voxels in each middle subsection after normalization (detrending and dividing by standard deviation) of each individual voxel time series. Circles mark estimates of the time series breakpoints. **(C)** Plot of change in estimated breakpoint between middle subsection averaged time series. Plotted times indicate change relative to the estimated breakpoint of subsection A. **(D)** Display of the averaged time series of voxels from the top, middle, and bottom sections of the carpet plot without normalization of the time series. **(E)** Display of the averaged time series of voxels from the top, middle, and bottom sections of the carpet plot after normalization (detrending and dividing by standard deviation) of each time series. Circles mark estimates of the time series breakpoints. **(F)** Plot of change in estimated breakpoint between bottom, middle, and top carpet plot averaged time series. Plotted times indicate change relative to the estimated breakpoint of the bottom section.

As discussed in the introduction, it has been shown that CO_2_-derived delay times computed *via* the cross-correlation method can be skewed by varying responses of the brain to the CO_2_ bolus arrival, with a primary issue being that the overall spread of delay times throughout all voxels can extend far beyond the expected time it takes for blood to flow through the whole brain (around 5–6 s) (Hoffmann et al., 2000). To further investigate this issue, we examine whether the shape of the voxel BOLD time series was associated with that voxel’s assigned cross-correlation delay time (and thus the voxel’s vertical location in the carpet plot). Voxel time series from the top, middle, and bottom portions of the carpet plot were averaged using two methods: first using the original, unfiltered voxel time series, and second where each individual time series was detrended, divided by the time series standard deviation, and frequency filtered (0.001–0.02 Hz). Finally, we more closely observe the time series of voxels in the middle of the carpet plot by dividing it into four stacked subsections, each section representing voxels grouped into 5-s windows based on their assigned delay time (recall that the characterization of the middle section is based on voxels which are assigned delay times within a 20-s window; see Figure 3 for visualization). These four subsections will be referred to as middle subsections A through D (from lowest to highest delay grouping). Voxel time series within each middle subsection were averaged (see example displayed in Figure 3) after normalization and frequency filtering. To compare the relative signal to noise ratio between carpet plot sections, roughly reflected in the magnitude of CO_2_-induced signal increase, the standard deviations of the bottom, middle, and top averaged time series (without filtering and normalization—see Figure 3D) were computed and compared using a Wilcoxon signed rank test with Bonferroni correction for multiple comparisons.

For the averaged time series of the top, middle, and bottom carpet plot regions, as well as that of each middle subsection, the breakpoint (i.e. time point when the time series signal begins to increase, or break from the baseline level, in correspondence with the arrival of the CO_2_ bolus) was estimated following a procedure similar to that proposed by Niftrik et al. (2017). First, an estimate of the breakpoint of the whole-brain averaged time series was computed based on the start time of the measured P_ET_CO_2_ signal increase (see Supplementary Section S3 for details). Then, for a given averaged time series from a carpet plot section, the baseline signal level was computed as the average signal intensity during 1 minute before the estimated whole-brain breakpoint. The peak signal intensity (i.e. the signal intensity during the second minute of the first bolus of elevated CO_2_) was estimated as the average signal intensity of the 1-min time span beginning 1 minute after the estimated whole-brain breakpoint. The breakpoint of the given time series was then computed as the time index when the time series first increased by 10% of the difference between the baseline and peak signal levels. The 10% breakpoint threshold was demonstrated by Niftrik et al. (2017) and is necessary to ensure that the computed time point reflects CO_2_-induced BOLD signal increase (as opposed to natural signal fluctuations). Breakpoint estimates are illustrated in Figure 3.

To investigate whether the top, middle, and bottom carpet plot regions were associated with particular brain regions, masks were created which specified which voxels commonly fell within each carpet plot section. These masks were computed by creating subject-specific section masks, registering these masks to the same standardized ICBM MNI-152 space (Kötter et al., 2001; Mazziotta et al., 2001), and keeping voxels which appeared in at least half of all subjects within the standardized space. In addition, subject-specific masks of voxels which showed little CO_2_-induced signal change (marked by absolute MCCC below 0.3) were created and registered to the standardized space to analyze common locations of such voxels. The carpet plot locations (top, middle, or bottom section) of such voxels were also noted.

### 2.7 Similarity comparison using structural similarity index (SSIM)

**FIGURE 4:**
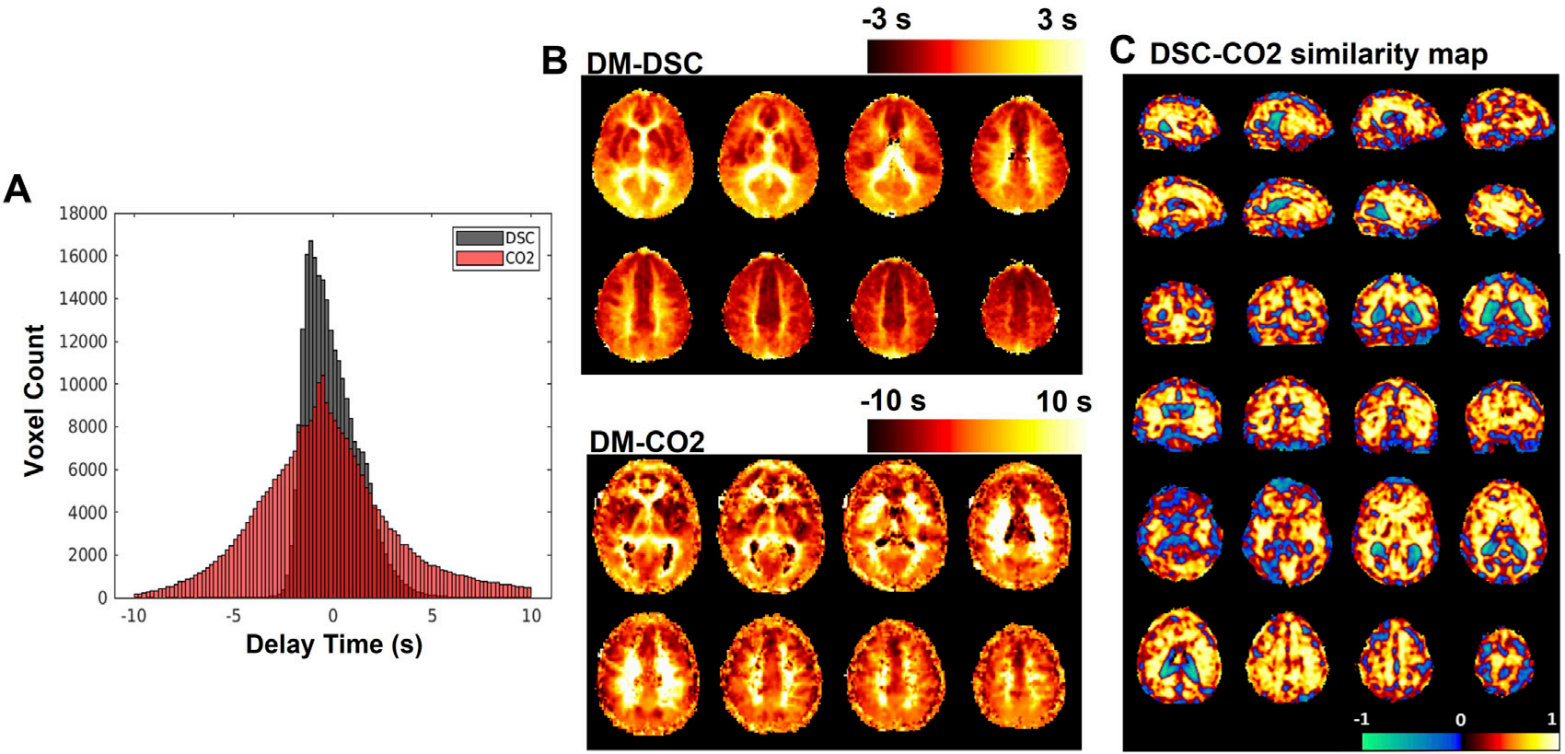
**(A)** Histograms of delay maps computed from DSC-MRI and CO_2_-challenge fMRI. **(B)** Sample slices of subject-averaged DSC-MRI and CO_2_-fMRI delay maps. **(C)** SSIM map displaying local (per-voxel) structural similarity s values comparing subject-averaged DSC bolus arrival delay map with the subject-averaged original CO_2_ arrival delay map. The structural similarity metric s can be interpreted similarly to Pearson’s correlation coefficient; values range from −1 to 1, where high positive values indicate higher structural similarity, lower negative values indicate inverted structural similarity, and values near zero represent little similarity.

We employed the structural similarity index measure (SSIM) to compare the similarity between the subject-averaged DSC-TTP map and the subject-averaged CO_2_-MRI delay map (Figure 4C). Unlike other common similarity metrics which only provide a single value indicating the similarity as a global assessment (such as the Pearson correlation), the SSIM provides spatial similarity information by calculating local statistics (Zhou et al., 2004). The two subject-averaged DSC and CO_2_ delay maps were demeaned and masked such that only voxels that existed in both delay maps were included. For 3D images, the SSIM algorithm utilizes a cubic window with a Gaussian weighting function to compute local image metrics during the computation, meaning that the program cannot compute an SSIM value for voxels near the brain edge if background voxels do not contain some value. To resolve this issue, which would cause the loss of a large number of edge voxels during the SSIM computation, we assigned the average delay value (i.e., zero) to all voxels containing no delay value (due to either being background voxels or the DSC-TTP program not returning a valid TTP delay, especially in ventricle regions). Note that while this choice can introduce bias to the structural similarity metric, the number of empty voxels inside the brain is very small and the choice to fill empty or edge voxels with the averaged delay value should minimize any introduced bias. The SSIM comparison was then conducted using the MATLAB command *ssim*. After the SSIM computation, those voxels filled with average delay (zero) previously were masked out.

The SSIM program computes a voxel-wise similarity map, where the resulting voxel-specific index value is computed using data from a specified cubic window surrounding the voxel. The SSIM algorithm incorporates three computed measurements: luminance, contrast, and structure. For the purposes of this study, only the “structure” element of the SSIM was used (see Discussion for more details regarding this choice). The structure element s of two compared cubic windows x and y is computed as:

$$s\left(x,y\right)=\frac{\sigma_{xy}+c}{\sigma_{x}\sigma_{y}+c}$$ (1)

The parameters of Eq. 1 are computed as:

$$\mu_{x}=\overset{N}{\underset{i=1}{\sum }}w_{i}x_{i}$$ (2)

$$\sigma_{x}={\left(\overset{N}{\underset{i=1}{\sum }}w_{i}{\left(x_{i}-\mu_{x}\right)}^{2}\right)}^{1/2}$$ (3)

$$\sigma_{xy}=\overset{N}{\underset{i=1}{\sum }}w_{i}\left(x_{i}-\mu_{x}\right)\left(y_{i}-\mu_{y}\right)$$ (4)

where c is a stabilizing constant and $w_{i}$ represents the weight assigned to the voxel according to the Gaussian weighting function (Zhou et al., 2004). The structure element is similar to a local estimate of Pearson’s correlation coefficient between the two windows with the added adjustment of the Gaussian weighting function. For the remainder of this paper, references to “structural similarity” will refer to this structure element of the SSIM. The default window size and weighting settings were used, resulting in a window size of 11 × 11 × 11 voxels and standard deviation of the Gaussian weighting function as 1.5. This program returns a structural similarity voxel map with index values ranging from −1 to 1. Negative values indicate inverted structure between the two images surrounding a specific voxel (Zhou et al., 2004). A higher magnitude value indicates higher similarity between two images in the region within the filter window surrounding the voxel. A global average structural similarity value was computed by averaging the resulting values over all brain voxels.

**FIGURE 5:**
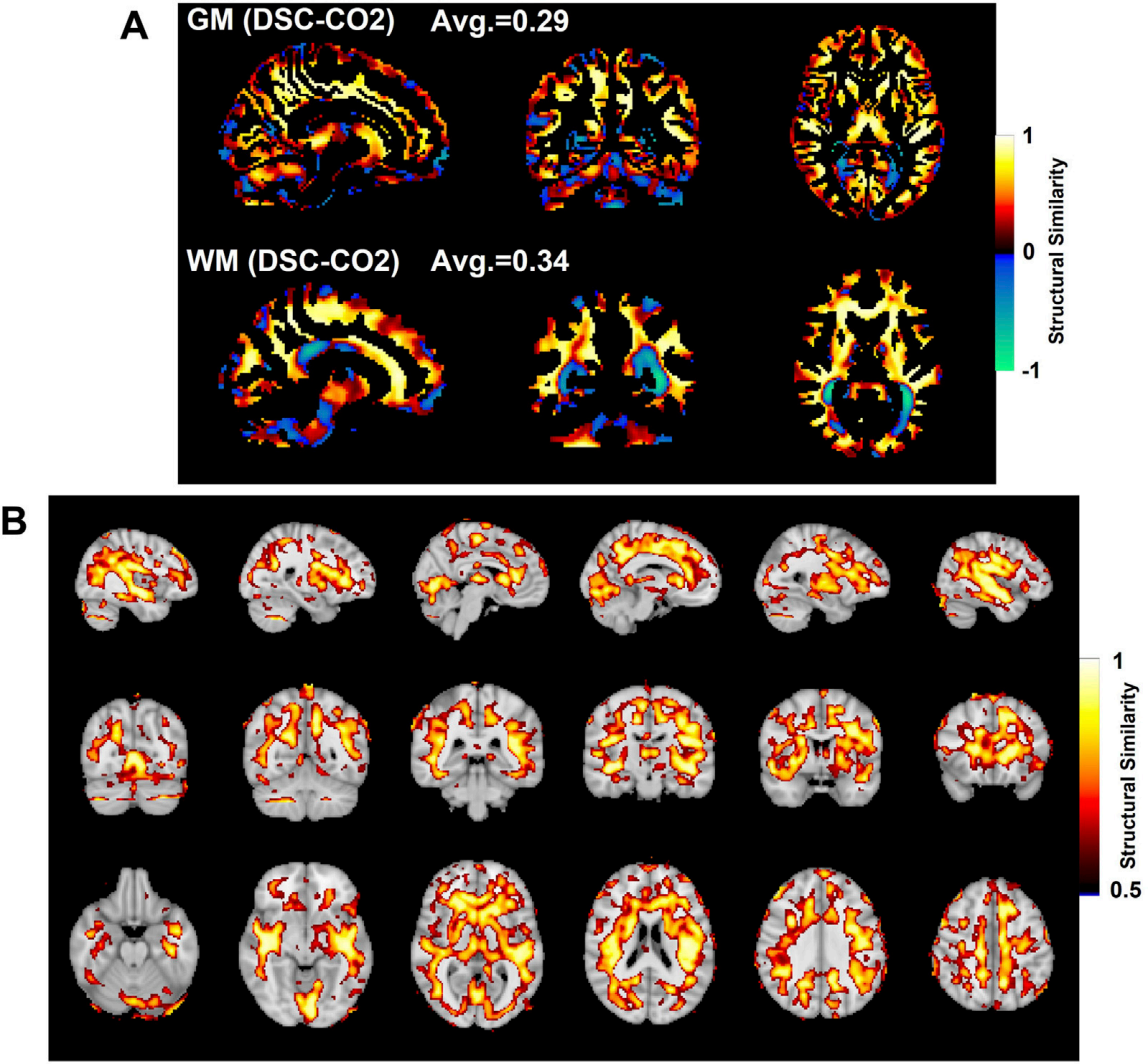
Comparison of SSIM values in gray matter (GM, top) and white matter (WM, bottom) shown in **(A)**. SSIM maps compare the subject-averaged DSC bolus arrival delay map with the subject-averaged CO_2_ arrival delay map. Voxels with SSIM values greater than 0.5 are shown in **(B)**.

**FIGURE 6:**
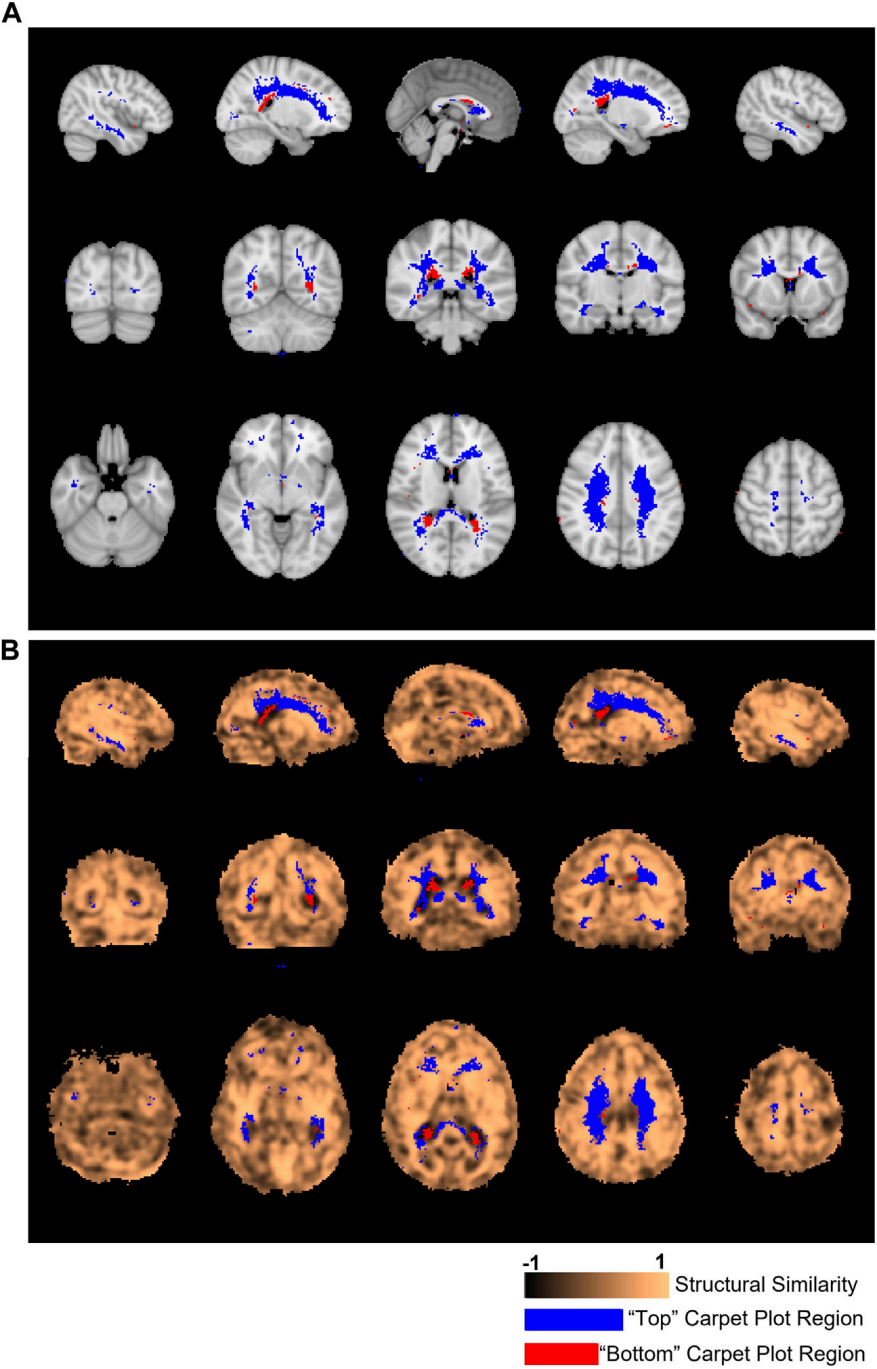
Locations of voxels located in top and bottom carpet plot regions and correspondence with structural similarity map. **(A)** Colored voxels indicate locations corresponding to voxels which were located in the top and bottom regions of CO_2_ carpet plots in at least half of all subjects. Blue voxels correspond to those cropped from the top of the carpet plot (with extra long CO_2_-derived delay times), while red voxels correspond to those cropped from the bottom of the carpet plot (with extra short CO_2_-derived delay times). **(B)** The same cropped carpet plot voxels from **(A)** are overlaid on the structural similarity value map comparing the subject-averaged DSC-TTP delay map with the subject-averaged CO_2_-arrival delay map.

Structural similarity values were averaged over GM regions and over WM regions, with these regions illustrated in Figure 5. In addition, structural similarity values were averaged over the previously detailed carpet plot section masks, which isolate the voxels which commonly belong to the top, middle, and bottom carpet plot regions. A comparison of the top and bottom carpet plot region masks with the computed structural similarity map is shown in Figure 6.

### 2.8 Statistical analysis

To evaluate the association between computed breakpoints and the carpet plot section from which the time series was derived, Wilcoxon signed rank tests with Bonferroni correction for multiple comparisons were performed comparing breakpoints from adjacent carpet plot sections. Wilcoxon rank sum tests (with Bonferroni correction when applicable) were applied to evaluate the differences in SSIM values in the three sections (top, middle, and bottom) of a carpet plot and in the GM and WM regions.

## 3 Results

### 3.1 Transit times derived from CO_2_ carpet plots

Carpet plots for all subjects are displayed in Figure 2. CO_2_ bolus transit times were computed as the horizontal distance, measuring time, of the sloped carpet plot edge (displayed in red). The detected CO_2_-arrival edges had transit times of 5.32±1.04 s (mean±s.d.). Dividing lines between the top, middle, and bottom carpet plot regions are shown in Figure 2 in blue. On average, the middle carpet plot section contained 77.8±5.4% of all voxels.

### 3.2 Carpet plot analysis results

Figure 3 displays sample results of the carpet plot analysis for one subject. Figure 3A illustrates the breakdown of the carpet plot into three regions—“top”, “middle”, and “bottom”—and illustrates the breakdown of the middle region into four smaller subgroups—middle subsections A, B, C, and D. We note that within the middle region, subsections B and C contain far more voxels than subsections A and D, since subgroups are groups of voxels with assigned delay times within a 5-s window. The distribution of delay times is primarily bell-shaped, meaning there are more voxels with assigned delay times close to the mean of the distribution. Figures 3B, D, E display the averaged time series of voxels for each grouping illustrated in (a) for the given sample subject. Figures displaying the same information in Figures 3B, D, E for all other subjects can be found in the Supplementary Figures S3–S5; the results derived from other subjects followed similar trends to those shown here.

Figures 3C, F illustrate the relationships between estimated breakpoints within the four middle subsections (C) and within the top, middle, and bottom carpet plot regions (F). These breakpoints represent an estimation of the true blood arrival time point for the voxel grouping, in contrast to the skewed arrival delay time assigned *via* cross-correlation. Analysis of these breakpoints provides an evaluation of whether voxels assigned a later venous blood arrival time *via* cross-correlation truly have a later arrival time as estimated *via* the breakpoint. This analysis was conducted on averaged subsections of voxels because computation of voxel-wise breakpoints resulted in unsatisfactory quality in the breakpoint estimates, likely due to noisiness in individual voxel time series (this is further discussed in the Discussion). The breakpoints of the middle subsections (from subsection A to D, i.e., from lower to higher delay times) are non-decreasing and demonstrate a positive association between the assigned delay time for the grouping and the breakpoint of the grouping’s averaged time series. A statistically significant (Wilcoxon signed rank test, *p* < 0.025) difference is found when comparing the bottom breakpoints with the middle breakpoints, as well as the middle breakpoints with top breakpoints. A statistically significant difference (Wilcoxon signed rank test, *p* < 0.016) was found in comparing middle subsections C vs. D. For the raw averaged time series, illustrated in Figure 3D, the average standard deviation (computed as a simple metric to compare the relative signal-to-noise ratios between carpet plot sections) of the middle averaged time series signal across subjects was 154.3 (a.u.), which is significantly higher (Wilcoxon signed rank test, *p* < 0.025) than both the averaged standard deviations of 41.6 and 46.7 for the top and bottom averaged time series, respectively. Similar differences in CO_2_-induced signal strength are seen in the normalized and filtered averaged time series shown in Figure 3E.

The percentage (mean ± s.d.) of voxels within the top, middle, and bottom carpet plot regions reflecting low (below 0.3) absolute MCCC were found to be 8.97% ± 4.25%, 0.85% ± 0.53%, and 13.15% ± 5.21%, respectively. Subject-specific masks of the brain locations of such voxels were created and analyzed, but the resulting masks contained very few, sporadic voxel locations which reflected little similarity between subjects; thus no further details on these results are given.

### 3.3 Structural similarity map with SSIM and regional comparison of SSIM

The subject-averaged delay maps computed from DSC-data and CO_2_ challenge data are displayed in Figure 4B, with histograms displayed in Figure 4A. In addition, Figure 4C shows the similarity map (voxel-wise s as defined previously) acquired from comparing the averaged DSC-TTP map with the averaged BOLD-CO_2_ MRI delay map *via* the SSIM structure element. For the DSC-CO_2_ delay map comparison, the whole-brain average structural similarity value $\bar{s}$ is 0.28.

Figure 5A shows the SSIM value similarity maps for the DSC-CO_2_ delay maps comparison broken down into GM and WM regions. The average SSIM values for each map and region are also displayed above each image. The GM and WM regions produce similar averaged SSIM values (0.29 for GM and 0.34 for WM; medians statistically different based on Wilcoxon rank sum test, *p* < 0.05). 30% of the voxels in the DSC-CO_2_ similarity map were found to have relatively high (greater than 0.5) SSIM values and are displayed in Figure 5B. The percentage of relatively high SSIM values in GM and WM is 29% and 40%, respectively. Rough analysis of the presence of different tissue types in the three carpet plot sections suggested that the top carpet plot regions consisted of mostly WM, the bottom carpet plot regions contain a large number of voxels classified as neither GM or WM, and the middle carpet plot regions contain a roughly equal amount of GM and WM (see Supplementary Table S2 for further details).

Figure 6A displays the location of the top and bottom carpet plot region masks, which represent areas of the brain that were assigned to either the top or bottom carpet plot regions in at least half of subjects. These same voxel masks are overlaid in Figure 6B with the structural similarity map comparing the subject-averaged CO_2_ delay map with the subject-averaged DSC delay map. The average structural similarity s within the top, middle, and bottom carpet plot regions were 0.36±0.41, 0.32±0.32, and −0.21±0.41, respectively. Significantly different distributions in s values were found between all three carpet plot regions (Wilcoxon rank sum test, *p* < 0.01 for all three comparisons).

## 4 Discussion

In this study, we use carpet plots and related analyses to assess the issue of widely spread venous blood arrival delay values assigned *via* the cross-correlation method applied to CO_2_-fMRI. Results (Figure 4A) indicated, as expected, that the venous blood arrival time values computed using CO_2_-challenge fMRI produce a wider range of delay values than those derived from DSC-MRI. Carpet plot analysis yielded an average estimated blood transit time of 5.32 s. The analysis of averaged time series from different groups of voxels, identified in part using the carpet plot, illustrated patterns in the shape of voxel time series based on a voxel’s assigned delay time, which we will further discuss in this section.

### 4.1 Review of known inaccuracies in the cross-correlation method

**FIGURE 7:**
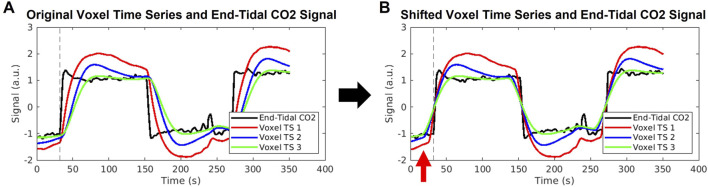
Illustration of errors stemming from cross-correlation method of delay times. **(A)** An example end-tidal CO_2_ time series is shown in black, with three example voxel time series shown in red, blue, and green. These voxel time series are created by convolution of the black end-tidal CO_2_ time series with three different hemodynamic response functions representative of different areas of the brain, as used in Yao et al. (2021). The common break point for all signals is illustrated by the dashed vertical line. **(B)** The voxel time series are shifted according to the maximum cross-correlation method, which aligns the signals such that maximum correlation with the end-tidal CO_2_ time series is achieved. The same end-tidal CO_2_ breakpoint as in **(A)** is shown by the dashed vertical line. The red arrow highlights that the voxel time series now display breakpoints occurring before the end-tidal CO_2_ increase, which is not possible. The red, blue, and green voxel time series were shifted to the left by 7, 16, and 19 s, respectively, which represent unrealistically high delay values.

We first wish to clearly state the inaccuracy associated with computing venous blood arrival times from CO_2_-challenge fMRI data using the method of maximum cross-correlation of each voxel time series with some version of the inhaled CO_2_ signal. It has been previously demonstrated that the maximum cross-correlation method can yield a blood-arrival delay span of 20.1 s across the whole brain (Niftrik et al., 2017), which is inconsistent with the fact that the cerebral blood transit time is approximately 5–6 s (Hoffmann et al., 2000). To further illustrate this error stemming from cross-correlation, we cross-correlated the CO_2_ measurement with three BOLD signals with different shapes while sharing the same breakpoint (see Figure 7). These three BOLD signals (in red, green, and blue) simulate voxels having the same arrival time of the elevated CO_2_ (the same breakpoint), but with different response times to the elevated CO_2_, and were computed by convolving the true P_ET_CO_2_ measurement with three different hemodynamic response functions from Yao et al. (2021). Depending on the extent of the distortion of the BOLD waveform from the CO_2_ measurement, a delay time offset could be as high as 19 s. This delay time offset is not due to the real CO_2_ arrival offset but the brain’s CO_2_ response behavior. Therefore, the delay time calculated for the CO_2_-fMRI by the cross-correlation method can be skewed by this CO_2_ response behavior, raising the question of whether arrival times computed *via* cross-correlation truly reflect the venous blood arrival time or are purely dominated by the CO_2_ response behavior of the voxel. We address a part of this issue by demonstrating that analyzing CO_2_-fMRI data using carpet plots can lead to improved estimates of the cerebral blood transit time. We also use voxel groupings suggested by inspection of the carpet plots to better understand the relationship between signal shape and assigned delay time.

### 4.2 Transit times

The slope detection method introduced in our previous study (Fitzgerald et al., 2021) was applied to the middle sections of carpet plots to compute blood transit time through the associated brain voxels, yielding an average estimate of 5.32 s. This observation of a linear pattern in the middle region is important because we observe this linear pattern in nearly the entire carpet plot (over 95% of voxels) for carpet plots derived from DSC-MRI; such carpet plots are illustrated in our previous paper (Fitzgerald et al., 2021); Supplementary Figure S6. The definition of the middle carpet plot section is such that it contains voxels with delays within a 20-s window, meaning that the cross-correlation method alone would suggest a 20-s blood transit time through this region of voxels. An average of 5.32 s represents an improved transit time estimate that moves much closer to the expected 5–6 s. We note that the middle carpet plot sections contained, on average, about 78% of voxels in the brain, meaning that about 22% of voxels were not directly represented by this estimated transit time. This implies that these estimated transit times could be underestimates, primarily if the voxels located in the top and bottom sections truly do experience blood arrival after (for top voxels) or before (for bottom voxels) those in the middle section. As will be discussed, our analysis suggests that voxels within the top of the carpet plot, though they do have assigned delays that are likely skewed too long, are likely to be roughly correctly assigned sequentially, strengthening the likelihood that these transit times are underestimates of the whole-brain blood flow. However, increasing the estimated transit times by 22% (proportionally to the number of voxels not represented by the middle region) would result in an average transit time of 6.44 s, which is still much improved compared to the 20-s spread implied by the cross-correlation method alone. In addition, the middle carpet plot sections appear to be composed of roughly equal numbers of both GM and WM voxels, suggesting that the computed transit time does not reflect blood passage only through GM portions of the brain.

Other studies have presented similar methodologies for computing whole-brain blood transit times based on BOLD MRI signals, yielding similar results. Aso et al. (2020) computed voxel-wise lag (i.e., delay) maps based on cross-correlation of resting state voxel BOLD time series with an extracted seed time series intended to represent the center of the vascular tree. This was followed by computation of global transit time as the sum of estimates of the arterial transit time (computed as the average of voxels with positive lag times relative to center) and the venous transit time (computed as the average of voxels with negative lag times relative to center), with the majority of global transit time estimates falling into the range of 4–6 s (Aso et al., 2020). Additionally, Bhogal et al. (2022) implemented a carpet method for computing global transit times using controlled hypoxia as a source of BOLD contrast, yielding an average global transit time of 4.5 s. Similar investigation of using hypoxia to induce BOLD contrast has been explored in (Sayin et al., 2022). These alternative approaches demonstrate the feasibility of BOLD signal-based methods for computing global transit time which do not rely on induced hypercapnia.

### 4.3 Analysis of signal behavior in carpet plot regions

Figure 3 illustrates several behaviors and traits of the voxel time series within different sections of the carpet plots. First, Figures 3D, E demonstrate that the averaged time series derived from the middle of the carpet plot tends to have a more profound signal-to-noise ratio than that of the top and bottom carpet plot regions, which implies that voxels within the middle carpet plot region tend to experience a greater degree of signal change due to the arrival of the CO_2_ bolus. Observing this difference in induced signal change after normalizing (detrended and divided by standard deviation) voxel time series, as in Figure 3E, demonstrates that a higher percentage of voxels within the top and bottom carpet plot regions likely display little to no CO_2_-induced signal change (causing the averaged CO_2_ signal strength to be weaker). One possible reason could rely on the intrinsic hemodynamic responses in some brain regions which tend to have reduced BOLD response to the same CO_2_ stimulus, measured by reduced cerebrovascular reactivity (Bhogal et al., 2015; Niftrik et al., 2017). A more detailed discussion regarding the main location of top and bottom carpet plot regions and the effect on the signal behavior can be found in the next section.

In addition, it can be observed that the averaged time series from the top carpet plot section displays a notably different signal shape than that of the middle section, in that the CO_2_-induced signal rise and fall is much sharper in the middle section. To help quantify this difference in signal shape, we estimated two additional metrics from the signals: the time index when the increasing CO_2_ signal reached 90% of the difference between baseline and peak signal intensity (called “peak point” here), and the slope of the signal in between the estimated breakpoint and peak point. Details regarding the computation of these metrics and the associated results are listed in Supplementary Figure S7. This analysis showed that voxels in the top of the carpet plot, thus having been assigned very long delay times, do tend to have signal shapes which reflect slower rise and fall times. This is exactly the signal behavior which is demonstrated in Figure 7 to lead to excessively long delay times in the cross-correlation method. Similarly, this trend of higher delay times associated with slower responses to the CO_2_ bolus can be seen in the middle carpet plot subsections illustrated in Figure 3B. Peak points and slopes for these averaged time series were also computed and demonstrated that longer delay times were associated with lower slopes and longer peak points (see Supplementary Figure S8).

These results demonstrate that, as is suggested by the discussion of Figure 7, the delay time assigned *via* cross-correlation in CO_2_-fMRI is largely associated with the shape of the voxel time series, which is dependent not only upon the true arrival time of the CO_2_ bolus, but also upon how quickly and how strongly a given brain region responds to the arrival of this CO_2_ bolus. It has been found that various brain regions respond to the CO_2_ stimuli differently (slow vs. rapid response) (Golestani et al., 2015; Poublanc et al., 2015; Fisher et al., 2017; Prokopiou et al., 2019), resulting in a variety of shapes of the BOLD waveform, which increases the difficulty of obtaining accurate delay times. The rise in signal intensity is not a result of detecting CO_2_ directly, but rather is the result of increased blood flow caused by vasodilation due to the increased CO_2_ presence. This raises an important question: does a delayed/weaker reactivity to the arrival of CO_2_ necessarily correspond to an actual delay in the CO_2_ arrival? To address this question, we computed estimates of the breakpoints of averaged time series from each carpet plot section, as displayed in Figures 3C, F. Estimating these breakpoints, as described in Niftrik et al. (2017), could serve as a better estimate of the time point of CO_2_ arrival, given the known shortcomings of the cross-correlation method. Results indicated that on the averaged time signals, breakpoints do increase as the assigned delay values corresponding to the given carpet plot section increase. We note that the average change in breakpoint time between the averaged time series for middle subsections A and D was 3.75 s. Given that these four subsections were broken up according to 5-s delay windows, the cross-correlation method alone would suggest a range of delays in the realm of 15 s between the midpoints of subsections A and D. Thus the breakpoint computations show a tighter estimation of the change in venous blood arrival time, though it must be highlighted that this tighter estimation only comes through breakpoint computation on averaged BOLD time series over many voxels as opposed to individual voxel breakpoint estimates (discussed later in the Discussion). These observations provide a first point of support for the notion that the sequence of delay values is somewhat reliable despite the heavy influence of signal shape on the computed delay time. To further assess this question, we turn to analyzing the structural similarity between the subject-averaged CO_2_ and DSC delay maps.

### 4.4 DSC-MRI and CO_2_-MRI delay maps comparison

The structural similarity metric s is computed somewhat like a weighted correlation coefficient between data within the two compared windows centered around a given voxel. It can provide a rough intuition for whether the structure, or sequence of assigned delay values, is similar between the two windows. In observing the structural similarity map results, we first observe the regions with relatively high structural similarity values ( s greater than 0.5), as illustrated in Figure 5B. 30% of all brain voxels yielded s values greater than 0.5. Those voxels are mainly clustered in the temporal lobe, parietal lobe, occipital lobe, and WM. We note that regions of WM contained higher structural similarity values, on average, than areas of GM (Figure 5A). It is important to note, however, that since the structural similarity values s are computed using information within an 11 × 11 × 11 voxel Gaussian-weighted window around the central voxel, meaning that each s value reflects the similarity within a window around the voxel, possibly from multiple tissue types.

#### 4.4.1 Top of carpet plot

One of the primary issues of the maximum cross-correlation method applied to CO_2_-MRI is the resulting wide spread of CO_2_-derived delay values. To better understand this spread, we analyzed which voxel locations corresponded to those cropped from the top of CO_2_ carpet plots (indicating extra long CO_2_-derived delay values) and voxel locations which corresponded to those cropped from the bottom of CO_2_ carpet plots (indicating extra short CO_2_-derived delay times). These voxel locations are displayed in Figure 6. Observation of these voxel locations reveals that voxels with extra long CO_2_-derived delay times were primarily located in areas of deep WM in the brain. This also explains the observation that these voxels have lower signal-to-noise ratio compared with those in the middle carpet plot portion. This pattern of longer delays and lower signal-to-noise ratio in areas of deep WM is consistent with the results observed in the DSC-MRI data and in previous papers (Thomas et al., 2013b; Bhogal et al., 2015; Poublanc et al., 2015; Bhogal, 2021; Poublanc et al., 2021). Further, the observed BOLD signal pattern in each voxel is impacted by both the arrival of CO_2_ to the voxel and the blood vessel dilation which occurs due to CO_2_ being a vasodilator. Some studies (Bhogal et al., 2015; Niftrik et al., 2017) suggest that cerebrovascular reactivity, a measure of the magnitude of the response of brain regions to the effects of CO_2_ arrival, tends to be lower in regions of deep WM, which could suggest that the extra long CO_2_-derived delays observed in these regions in CO_2_ delay maps could also be influenced by low cerebrovascular reactivity in those areas. Additionally, results from Bhogal (2021) confirmed that reactivity in WM is notably different than that of GM and showed that the response of WM is also influenced by venous draining topology. Despite these sources of potential confounding of the assigned delay time within these voxels, analysis of the structural similarity map within the brain region mask associated with the top of the carpet plots (see Figure 6B) revealed that these voxels had equal, possible higher, structural similarity compared with those from the middle of the carpet plots. This suggests that these voxels with very long CO_2_-derived delays have similar reliability in their ordering with those from the middle of the carpet plot (whose delay times are expected to be more reliable) in terms of structural similarity to the DSC delay map.

#### 4.4.2 Bottom of carpet plot

Voxels which were removed from the bottom of the carpet plot were primarily located in regions near the boundary between WM and the lateral ventricles, particularly toward the posterior side of the brain. These voxels correspond to locations where the computed delay value was more than 10 s earlier than the average CO_2_-derived delay time across the brain, which likely cannot reflect true arrival of the CO_2_ given that the increased arterial CO_2_ would not likely occur so early in these specific voxels. We also note that these voxels at the bottom of the carpet plot were more likely to have BOLD time series which were negatively correlated with the global average time series. Analysis of the correlation values obtained during the cross-correlation delay computations revealed that voxels with negative correlations appeared in the bottom, middle, and top carpet plot regions at rates of 35%, 2%, and 21% on average, respectively.

Such observations (namely, of negatively correlated BOLD signals near the edge of the ventricles and with signal changes earlier than most voxels across the brain) align with previous studies (Bianciardi et al., 2011; Thomas et al., 2013a; Bright et al., 2014) which suggest that negative BOLD signal correlations are predominantly due to blood volume change instead of cerebral blood flow (CBF) increase. This volume change is due to dilation of ventricular vessels accompanied by shrinkage of cerebrospinal fluid (CSF) space, resulting in signal decrease which could overpower any BOLD signal increase. This hypothesis also explains the very short CO_2_-derived delays calculated for voxels with negatively correlated BOLD signals since CSF shrinkage could occur prior to the arrival of the CO_2_. Alternatively, another mechanism which could cause negatively correlated BOLD signals is the cerebral “steal effect”, in which multiple CBF changes (both increased and decreased CBF response in regions in and surrounding the voxel) simultaneously occur in response to the arrival of the CO_2_, meaning that regions with reduced CBF are compromised due to the increased CBF in other regions (Brawley, 1968; Poublanc et al., 2013; Sobczyk et al., 2014). Finally, one might hypothesize that the Bohr effect (Riggs, 1988), under which increased concentration of CO_2_ in the blood causes a decreased oxygen binding affinity in hemoglobin (thus increasing concentrations of deoxyhemoglobin), could theoretically cause a BOLD signal decrease in response to elevated blood CO_2_. However, the magnitude of any such signal changes due to the Bohr effect is expected to be very small. This claim is supported by the fact that an increase of 10 mmHg from baseline in end-tidal CO_2_ should only cause a very small shift in the oxyhemoglobin dissociation curve for normal blood partial pressure of O_2_ (PO_2_) (Levitzky, 2013), where normal arterial PO_2_ typically falls within the range of 75–100 mmHg (Ortiz-Prado et al., 2019). Further investigation is still needed to definitively explain this phenomenon of certain voxels displaying decreased BOLD signal in response to the CO_2_ bolus. As demonstrated in Figure 6B, the voxels with very short CO_2_-derived delay values corresponded with areas of the SSIM value map which were particularly low, indicating that these unrealistically low delay times are likely one of the main factors which differentiates the CO_2_ delay maps from the DSC delay maps.

As an added evaluation of the similarity between CO_2_ and DSC delay maps, CO_2_ carpet plot were reconstructed by sorting voxels according to the averaged DSC delay map (after registration to the subject’s native space) and transit times were recomputed. Details on these results are shared in the Supplementary Material, along with the resorted carpet plots in Supplementary Figure S9. We note that this method of sorting carpet plots resulted in varying transit time values and did appear to significantly reduce the visual clarity of the three carpet plot sections that are apparent when sorting *via* the subject-specific CO_2_ delay maps. This result suggests a lack of similarity between DSC and CO_2_-derived delay map sequence should be noted. The difference between these two delay map sequence could be partially explained by the different underlying mechanisms of DSC and CO_2_ MRI, as DSC-MRI tracks the passage of the paramagnetic gadolinium-based contrast agent, inducing a local signal loss while not affecting the vascular tone or cerebral blood flow, whereas CO_2_ stimulus induces various complicated hemodynamic reactions, resulting in changes in the BOLD signal waveform. However, it must also be acknowledged that this transit time computation method (*via* slope detection on carpet plots) is highly dependent upon precise sorting of the carpet plot voxels for each subject. Since this experiment sorted individual subject carpet plots based on an averaged delay map derived from a separate cohort of subjects, such inter-subject differences may likely account for a significant portion of the differences in carpet plot sorting. Confirmation of the similarity of carpet plots and transit times derived from CO_2_ *versus* DSC delay maps would be best evaluated on a cohort where both DSC and CO_2_ MRI can be conducted on the same subjects.

### 4.5 Alternative methods and reasoning for chosen methodology

We wish to note two alternative methods for computing venous blood arrival times from CO_2_-MRI data and explain the observed shortcomings with these methods. First, the improved transit times derived from carpet plots suggest the possibility that the voxel-wise data points used to estimate the transit time edge may represent accurate CO_2_ arrival times. The carpet plot edge detection method, after applying an image smoothing filter, examines each image row (voxel time series) and identifies the time point of maximum point-to-point increase (signal derivative). These time points from all voxel time series in the carpet plot are then used to estimate the observed edge *via* linear regression. In one alternative method, these maximum signal derivative time points were computed as the voxel-wise venous blood delay times. When a subject-averaged delay map using this method was compared with the DSC-MRI delay map *via* SSIM comparison, the resulting brain-wise SSIM value was 0.20, indicating lower similarity than the cross-correlation delay map SSIM value of 0.28. Visual inspection of this delay map showed that it appeared “noisier”, or less smooth, than the delay map derived from cross-correlation.

Additionally, the discussion of Figure 3 would suggest that direct computation of the voxel time series “breakpoint”, or the starting point of the rise of the BOLD signal associated with arrival of the CO_2_ bolus, would yield a more accurate delay time than that of the cross-correlation method. This second alternative method was discussed and implemented in a study by Niftrik et al. (2017). However, implementation of this method on the data set discussed in this study failed to overcome the issue of delay-value distributions spanning a time window greater than the expected 5–6 s. One hypothesis for why these alternatives fail to out-perform the cross-correlation method is that these methods rely on direct observation of one specific portion of the voxel time series. These methods are thus very sensitive to noisiness in the voxel BOLD signal. In comparison, the cross-correlation method utilized information from the entire voxel time series, which contains two instances of rising and falling signal, which is helpful in overcoming noisiness of the signal (see Supplementary Section S8; Supplementary Figures S10–S12 for more details on alternative results).

We also wish to note the reasoning used in decisions regarding the use of the structure element of the SSIM for CO_2_-MRI vs. DSC-MRI comparisons. The traditional SSIM metric is computed as the multiplication of three terms: structure, luminance, and contrast (Zhou et al., 2004). The structure term, as described in this paper, reflects the degree of scaled covariance of the data within the two compared windows. The luminance term measures the similarity between the means of the two windows, while the contrast term measures the similarity in the variance between the two windows. For the purposes of this study, multiplying each of these terms as is done in the traditional metric made it difficult to interpret precisely what caused a low SSIM value, so we elected to analyze the structure, luminance, and contrast terms individually. It was found that the structure element was useful in that it was easily interpreted (due to similarity to Pearson’s correlation coefficient—see Eq. (1)), relatively insensitive to delay map normalization, and provided useful information reflecting regional similarity between the two delay maps. In contrast, the luminance and contrast elements were found to add relatively little additional information, with interpretation highly sensitive to data normalization methods (the choice of which is not trivial). Thus we chose to use the structure element as the main comparison metric for this study (see Supplementary Figure S13 for more details regarding this choice).

Another commonly used similarity metric, normalized mutual information (NMI), was also tested to evaluate the similarity between two delay maps. The NMI metric yields similarity values ranging from 0 to 1. The comparison of the spatial similarity maps derived from both metrics are shown in Supplementary Figures S14, S15. A very similar pattern of the similarity maps from both methods was observed in majority of the regions. However, the SSIM allows for highlighting inverted structure between the two maps through negative similarity values, while the NMI metric does not. This capability is the main reason the SSIM approach was chosen over the NMI approach.

### 4.6 Limitations and future directions

One limitation of this study is the limited number of subjects in the DSC-MRI (DSC scans on healthy subjects are rarely conducted due to the injection of the Gd contrast) and BOLD-CO_2_ MRI datasets, leading to a high variety of delay maps across subjects. Also, subjects in the CO_2_-MRI dataset and the DSC-MRI dataset are from different cohorts and not gender-matched, contributing to the inconsistency between delay maps derived from these two methodologies. These factors may lead to underestimation of the similarity between the averaged DSC-TTP map and the averaged BOLD-CO_2_ MRI delay map. Furthermore, in the CO_2_ arrival delay adjustment methodology, a linear fitting was assumed in the edge-detection algorithm, which might not work properly in diseased patients (e.g., Moyamoya, stroke). Non-linear fitting could be considered for such subjects. Additionally, the brain operates under different conditions during DSC-MRI and BOLD-CO_2_ MRI. DSC-MRI tracks the passage of the paramagnetic gadolinium-based contrast agent, which induces a local signal loss while not affecting the vascular tone or cerebral blood flow or volume. However, in BOLD-CO_2_ MRI, the CO_2_ stimulus induces various complicated hemodynamic reactions, which plays an important role in the BOLD signal changes. Analysis of averaged time series delays as divided by the carpet plot confirmed that the shape of voxel time series, characterized by the response of the voxel to the CO_2_ bolus arrival, are largely associated with the delay times assigned *via* cross correlation in CO_2_ challenge fMRI. An alternative strategy could be inducing hyperoxia (Moreton et al., 2016; Pinto et al., 2020) or hypoxia (Poublanc et al., 2021; Sayin et al., 2022) during BOLD imaging as an alternative gas stimulus to track blood flow with the benefit of less vascular reactivity changes during imaging.

A useful extension of this study would include an analysis of whether the carpet plot-based transit time computation method can be useful for quantifying blood transit times throughout localized subsections of the brain. Additionally, it may be possible to extend this methodology to use the fitted carpet plot edge line to estimate improved voxel-wise blood arrival delay times. To support such an extension, more work would need to be conducted to conclude whether the rank (i.e., ordering) of voxels in the carpet plot is truly accurate when computed based on the cross-correlation method.

## 5 Conclusion

In this study, we proposed a novel carpet plot-based method to reduce the estimated cerebral transit times derived from hypercapnia fMRI in healthy subjects and spatially compare the resulting delay maps with the DSC-MRI TTP maps. We demonstrated that, at least at a broad level, the sequence of voxels implied by the assigned CO_2_ delay values is still similar to, though not a perfect representation of, that derived from DSC-MRI. The tilted edge in the middle CO_2_ carpet plot regions mimics that observed in DSC-MRI carpet plots. Voxels in the top portion (extra long delays) were located in deep WM while those in the bottom portion were located in the periventricular region. The structural similarity (with DSC delays) of voxels associated with the top and middle carpet plot regions was shown to be positive and similar between the top and middle regions. Voxels falling within the bottom of the carpet plot were shown to have delay values that are unrealistically early and, on average, have poor (negative) structural similarity with the same voxels assigned delays *via* DSC-MRI. However, delay values of voxels in the top portion were largely affected by the hemodynamic response under CO_2_ challenge. Further research should focus on inducing alternative gas challenge to eliminate the vessel response, as well as collecting both DSC-MRI and gas challenge MRI data from the same cohort of subjects.

## Data Availability

The raw data supporting the conclusions of this article will be made available by the authors, without undue reservation.

## Abbreviations

BOLD, blood oxygen level dependent; CBF, cerebral blood flow; CSF, cerebrospinal fluid; DSC, dynamic susceptibility contrast; fMRI, functional magnetic resonance imaging; GM, gray matter; MCCC, maximum cross-correlation coefficient; MRI, magnetic resonance imaging; P_ET_CO_2_, partial pressure end-tidal CO_2_; SSIM, structural similarity index measure; TTP, time to peak; WM, white matter.
