# Supplementary material for: Using carpet plots to analyze blood transit times in the brain during hypercapnic challenge magnetic resonance imaging
Source: Front Physiol. 2023 Feb 15;14:1134804. doi: 10.3389/fphys.2023.1134804 (PMC9975721; doi:10.3389/fphys.2023.1134804)
Supplement: Supplementary file 1 [file DataSheet1.pdf]

Supplementary Material

Using Carpet Plots to Analyze Blood Transit Times in the Brain During Hypercapnic Challenge Magnetic Resonance Imaging

Bradley Fitzgerald[1†], Jinxia (Fiona) Yao[2†], Lia M. Hocke[3,4], Blaise deB. Frederick[3,4], Christiaan Hendrik Bas van Niftrik[5], and Yunjie Tong[2*]

[1]Elmore Family School of Electrical and Computer Engineering, Purdue University, West Lafayette, IN, United States

[2]Weldon School of Biomedical Engineering, Purdue University, West Lafayette, IN, United States

[3]McLean Imaging Center, McLean Hospital, Belmont, MA, United States

[4]Department of Psychiatry, Harvard Medical School, Boston, MA, United States

[5]Department of Neurosurgery, University Hospital Zurich, Zurich, Switzerland

[†]These authors contributed equally and share first authorship

*** Correspondence:**
Yunjie Tong
tong61@purdue.edu

1 Analysis of Effect of Middle Carpet Plot Size Parameter

When analyzing carpet plots, the middle carpet plot section was determined by selecting voxels which had CO_2 cross-correlation delay values which fell within a 20-second window centered at the median delay time. As stated in the manuscript, this window size was chosen based on empirical results with visual confirmation which suggested that such a window size served as a conservative but reliable method for isolating those voxels which followed the linear edge trend. For reference, Table S1 displays transit times for each subject computed using varying widths of this time window which determines the middle carpet plot region. As is expected, choosing a wider window includes a greater number of voxels within the middle carpet plot region and thus results in a longer transit time computation. Despite this, the computed times demonstrate that the transit time computed via this method are consistently much shorter than the window time width itself, demonstrating consistent improvement of the transit time estimate towards a reasonable value, as opposed to the excessively long transit time implied by the window time width.

Subject	Transit Time (s) for Given Window Time Width						
	12 s	16 s	20 s	24 s	28 s	32 s	36 s
1	1.98	2.58	3.07	3.64	4.14	4.69	5.28
2	3.51	4.56	5.41	5.77	6.22	6.46	6.46
3	4.52	5.49	6.83	7.81	8.60	9.87	10.44
4	3.72	4.47	5.37	6.28	6.97	7.59	8.2
5	3.66	4.57	5.38	6.02	6.58	7.29	8.21
6	3.50	4.79	5.74	6.65	7.37	7.91	8.46
7	3.66	4.45	5.29	6.07	7.46	9.32	10.92
8	4.16	4.95	5.44	5.89	6.31	6.60	6.80

Table S1. Transit times computed using various window sizes to determine the middle carpet plot region.

Additionally, here we present an initial proposal for an automated method for adapting this middle carpet plot window for each subject. In this method, the window size was first initialized as described in the main document (using a 20-second delay time window). A linear line was fit to the data as in the main document, and the slope of this linear fit recorded. To determine the final upper boundary of the window, the upper boundary was extended by 100 voxels and a new linear fit slope computed. If this new slope deviated by less than 1% from the original slope, the process was repeated. This was repeated until the new slope deviated by greater than 1% from the original slope, at which point the previous upper boundary was saved as final. A similar process was used to compute the final lower boundary.

Resulting carpet plot boundaries and transit times computed over the associated middle carpet plot sections are shown in Figure S1. Transit times computed using this method tended to be slightly larger than those reported in the main document, with an average of 6.98 +/- 1.28 seconds There is a statistically significant difference between these new transit times compared with the original reported transit times (Wilcoxon signed rank test, $p<0.01$). This increase is expected, as these newly

"adaptive" windows include a greater range of voxels. Although such an adaptive method offers increased flexibility in the middle carpet plot window size, such methods are still likely to rely on subjective parameters determined via empirical testing (in this case of this method, this parameter would be the 1% deviation threshold). Further effort and testing would be required to determine the optimal method for determining a flexible window size algorithm.

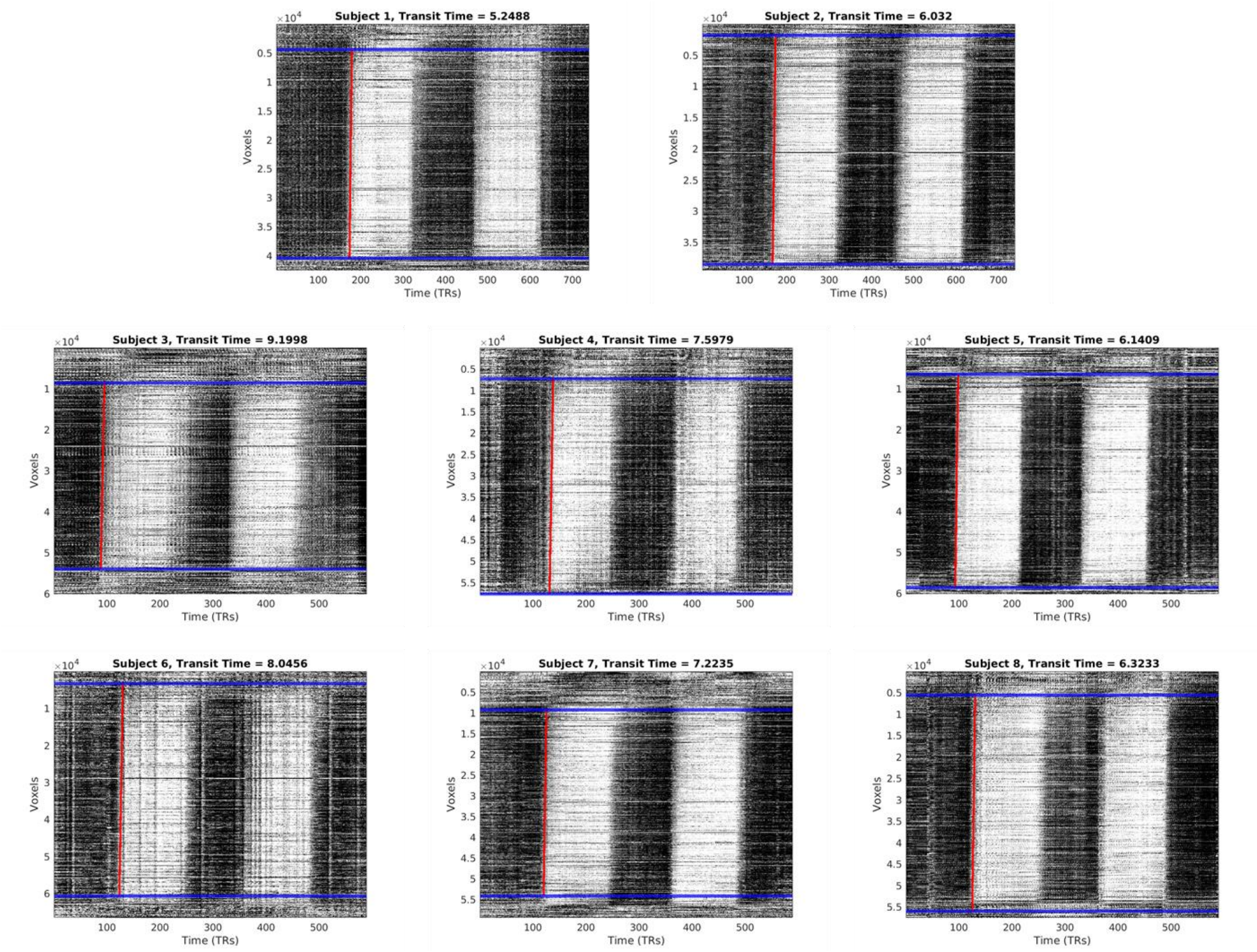

Supplementary Figure S1: Carpet plots and transit times derived using an adaptive window size algorithm which determined a unique middle carpet plot region (bounded by blue lines) for each subject. Detected edges are illustrated by red lines within the middle carpet plot region.

2 Transit Times for Second Carpet Plot Edge

Figure S2 displays the transit times computed for the carpet plot edge corresponding to the second CO_2 bolus. The average (+/- s.d.) transit time was computed to be 8.68 +/- 4.86 seconds. Transit

times computed for the second CO_2 bolus pass were not found to be significantly different than those reported for the first CO_2 bolus (Wilcoxon signed rank test, p>0.05).

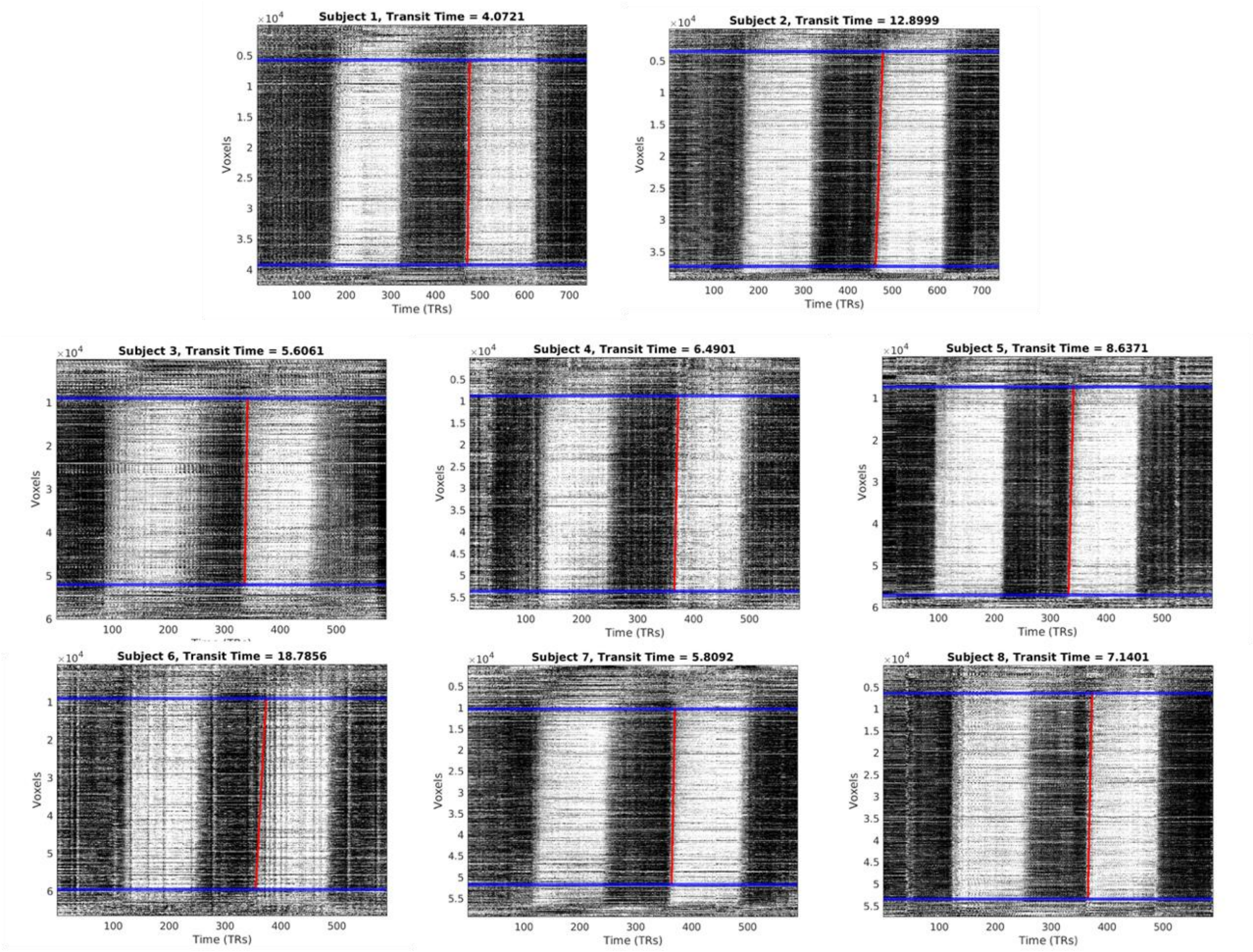

Supplementary Figure S2. Transit times computed for carpet plot edges corresponding to second CO_2 bolus.

3 Estimation of the Breakpoint for Whole-Brain Averaged Time Series

The breakpoint estimates for whole-brain averaged time series were computed as follows. First, a derivative filter was applied to the measured $P_{ET}CO_2$ signal (administered to the subject) and the time index of maximum derivative value was selected as the $P_{ET}CO_2$ breakpoint estimate. Observed BOLD signals in the brain do not immediately begin to rise at the same time as the $P_{ET}CO_2$; some time is needed for the elevated CO_2 levels to pass through the mechanical system, be inhaled by the subject, and travel through the subject's circulatory system, finally causing the elevated BOLD signal due to vasodilation caused by the elevated blood CO_2. To account for this delay, 10 seconds were added to the $P_{ET}CO_2$ breakpoint estimate, resulting in the whole-brain averaged time series breakpoint estimate.

4 Carpet Plot Averaged Time Series Data for All Subjects

Figures S3-S5 show the averaged time series data from various carpet plot subsections for all eight subjects, corresponding to Figure 3 from the main manuscript. Figure S3 shows averaged time series from the top, middle, and bottom carpet plot regions without signal filtering and normalization (corresponding to Figure 3d). Figure S4 shows these averaged time series with data normalization and filtering (corresponding to Figure 3e). Figure S5 shows the averaged time series from the carpet plot middle subsections (corresponding to Figure 3b).

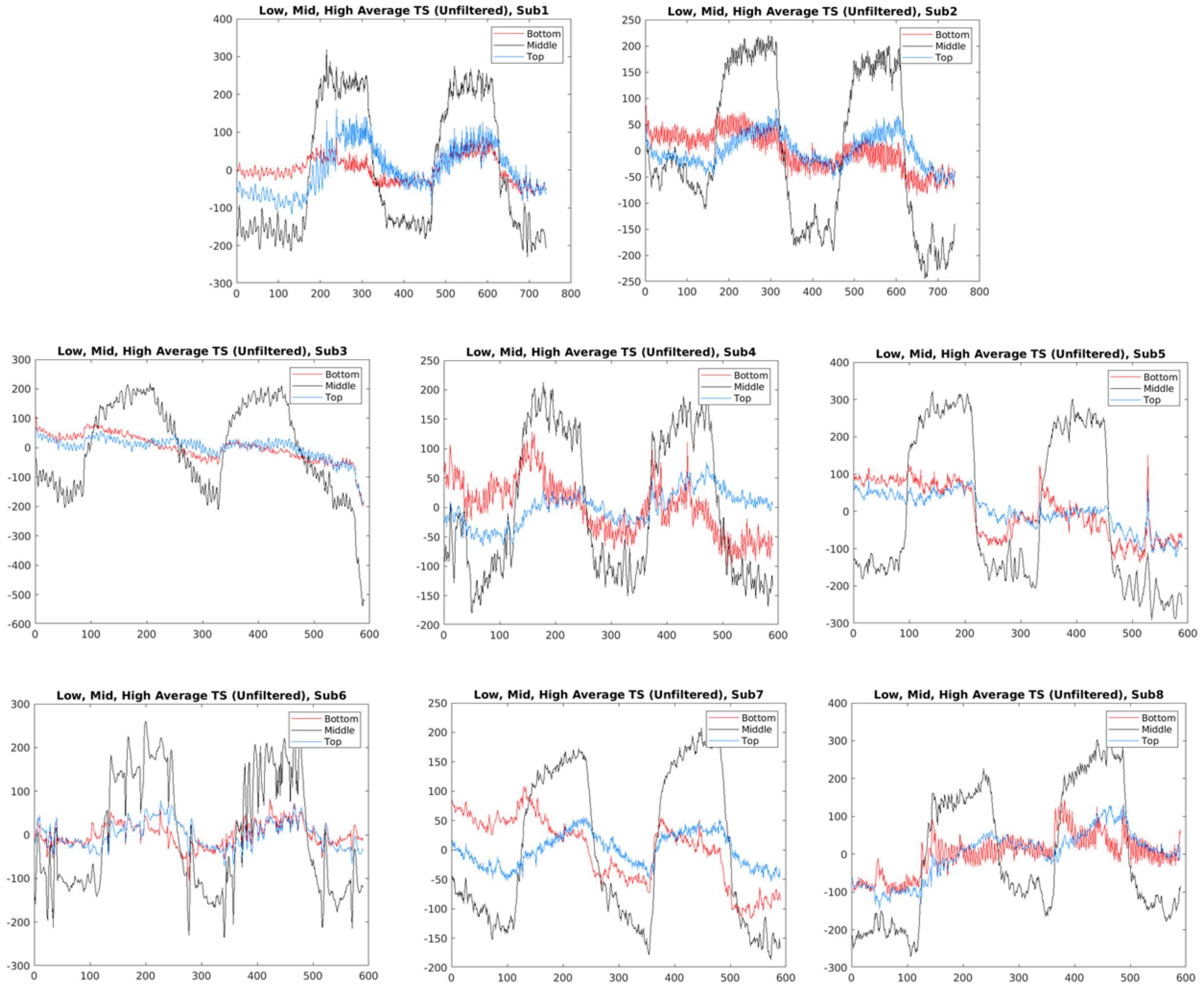

Supplementary Figure S3. Averaged time series of bottom, middle, and top carpet plot sections, without normalization and filtering, for all subjects.

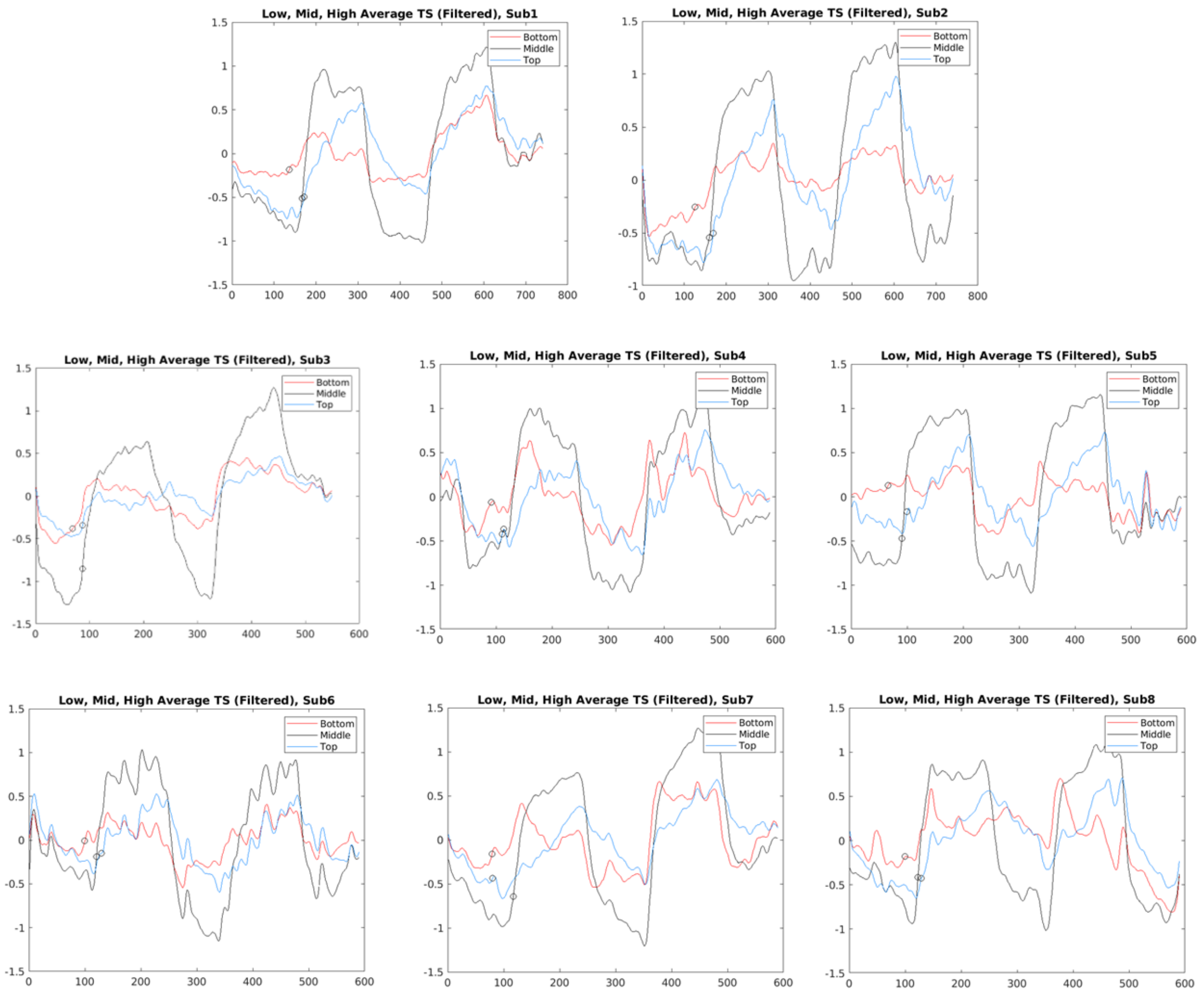

Supplementary Figure S4. Averaged time series of bottom, middle, and top carpet plot sections, after normalization and filtering, for all subjects.

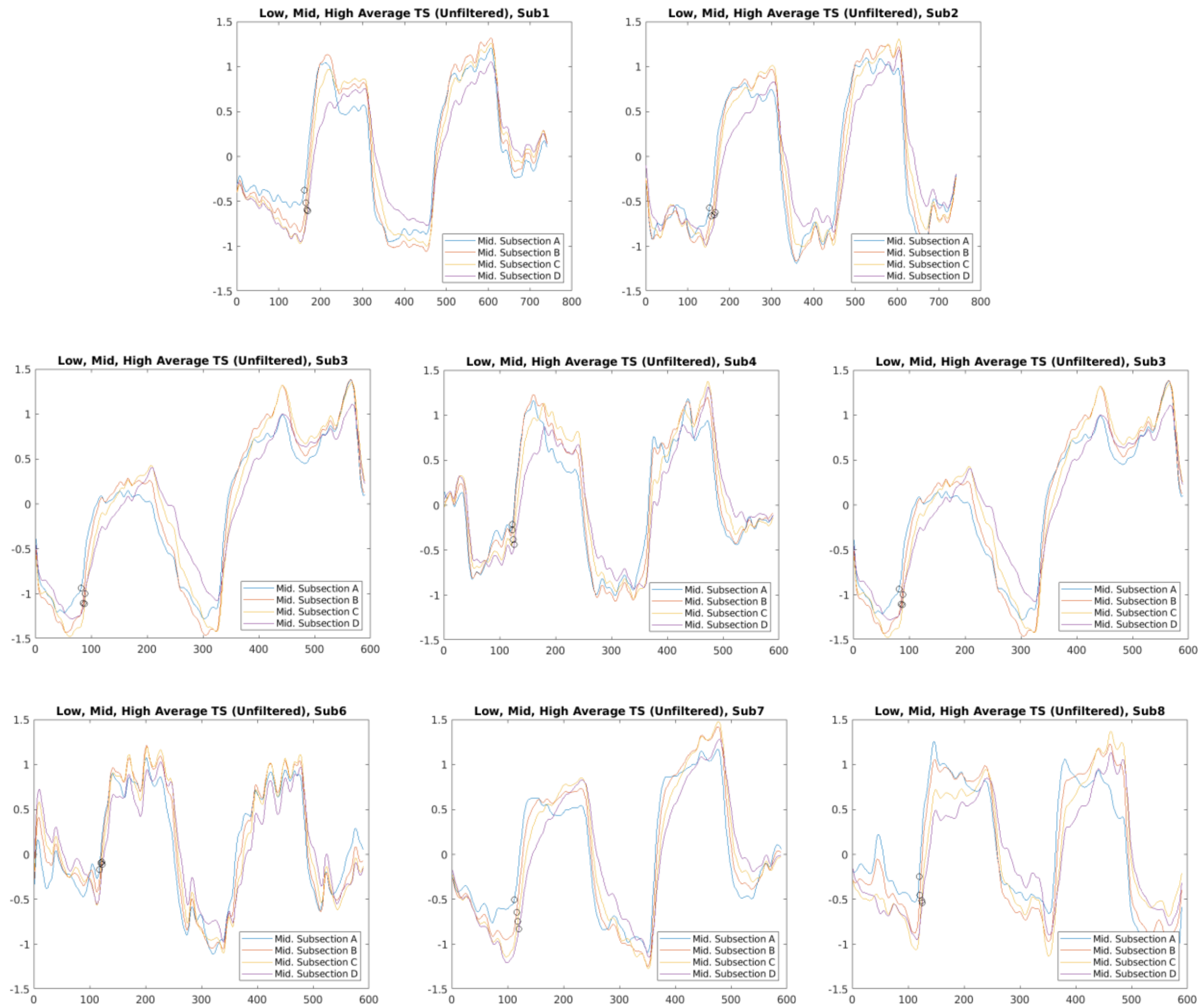

Supplementary Figure S5. Averaged time series of middle carpet plot subsections, after normalization and filtering, for all subjects.

5 Tissue Types Found in Each Carpet Plot Region

A rough analysis of tissue types was conducted by registering the ICBM-152 standardized GM and WM masks to individual subject spaces and measuring the percentage of GM and WM voxels which belonged to each carpet plot region (top, middle, and bottom). These percentages (reflecting the percentage of a given carpet plot region which was assigned to a particular tissue type) are shown in Table S2. Note that the fairly large portion of “Other” tissue type can be attributed to voxels which did not perfectly align with the GM and WM tissue masks even after registration, or those voxels which could not be classified confidently as either tissue type.

Tissue	Carpet Plot Region		
	Top	Middle	Bottom
GM	13.64 +/- 5.54 %	41.82 +/- 1.80 %	24.13 +/- 6.88 %
WM	57.74 +/- 6.03 %	37.57 +/- 1.49 %	32.66 +/- 7.13 %
Other	28.62 +/- 4.50 %	20.61 +/- 2.14 %	43.2 +/- 11.71 %

Table S2. Percentage (mean +/- s.d.) of each carpet plot region which was attributed to a given tissue type.

6 Carpet Plots Derived from DSC-MRI

Figure S6 shows the carpet plots constructed from gadolinium-based DSC-MRI data collected from a cohort of eight subjects (different subjects than CO_2-MRI subjects). Further details regarding these carpet plots can be found in our previous publication (Fitzgerald et al., 2021).

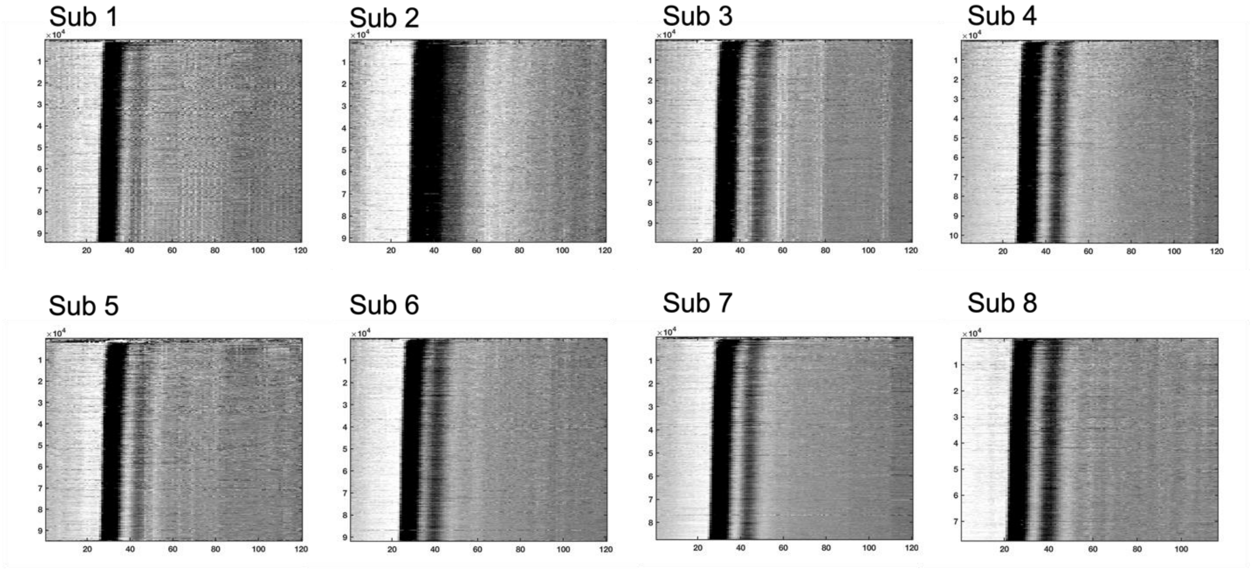

Supplementary Figure S6. The DSC-MRI carpet plots for all subjects.

Computation of Additional Signal Metrics and Results

The discussion of Figure 3 in the main document referenced the computation of several additional shape metrics when analyzing the behavior of voxel time series within various carpet plot sections. The purpose of this analysis is to demonstrate with quantitative evidence that the shape of the averaged time series from carpet plot sections with longer cross-correlation delay values was associated with signal shape involving slower signal rise and fall, which influences the performance of the cross-correlation delay assignment. Quantitative measurements of the signal shape were computed in the form of peak points, fall points, and upward slope. These measurements were

computed as follows: the baseline signal intensity was computed as the mean signal value within the 60 second window ending at the estimated break point. The peak signal intensity was computed as the mean signal value within the 60 second window beginning 60 seconds after the estimated break point (this corresponding to the second minute of CO_2-induced signal increase). The peak point was computed as the time index at which the rising CO_2-induced signal first reaches 90% of the difference between peak and baseline signal intensity. The fall point was computed as the time index at which the falling signal (caused by removal of increased CO_2) reaches the same point representing 90% of the difference between peak and baseline signal intensity. Finally, the upward signal slope was computed as the signal rise divided by time index span between the computed breakpoint and peak point. Results comparing these metrics for the bottom, middle, and top carpet plot regions is shown in Figure S7. We note that subject 3 had bottom and top average time series with particularly low signal-to-noise ratio, likely resulting in the unique behavior of subject 3 as shown in Figure S7. If subject 3 is removed, significant differences (Wilcoxon signed rank test, $p<0.025$ due to correction for two comparisons) were found in: bottom vs. middle fall points, and bottom vs. middle and middle vs. top rising slopes. These results indicate that higher cross-correlation assigned delay times are associated with the later time points at which the signal begins its descent and that the middle section voxels are associated with higher rates (slope) of signal increase.

Results comparing these metrics for the carpet plot middle subsections are shown in Figure S8. Significant differences between groups (Wilcoxon signed rank test, $p<0.016$ due to correction for three comparisons) were found for comparisons of A vs. B and B vs. C peak points, B vs. C and C vs. D fall points, and B vs. C and C vs. D upward slopes. This again demonstrates that higher cross-correlation assigned delay times are associated with the expected signal shape changes.

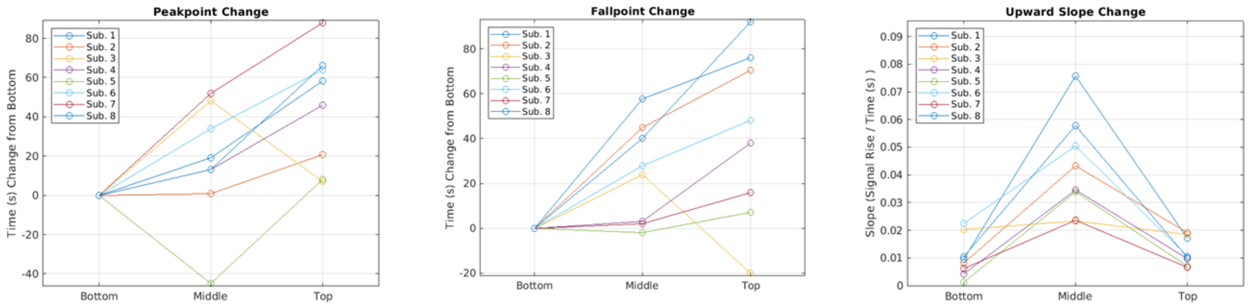

Supplementary Figure S7. Comparison of top, middle, and bottom carpet plot sections through various shape metrics. (Left) Change in peak point (time index, during CO_2-induced signal rise, at which the time series reached 90% of the difference between baseline signal value and mean peak signal value) relative to the bottom carpet plot section. (Center) Change in fall point (time index, during signal fall due to lowered CO_2, at which the time series lowered to 90% of the difference between baseline signal value and mean peak signal value) relative to the bottom carpet plot section.

(Right) Slope of averaged time series between the breakpoint and the peak point (i.e. slope of CO_2-induced signal rise in averaged time series).

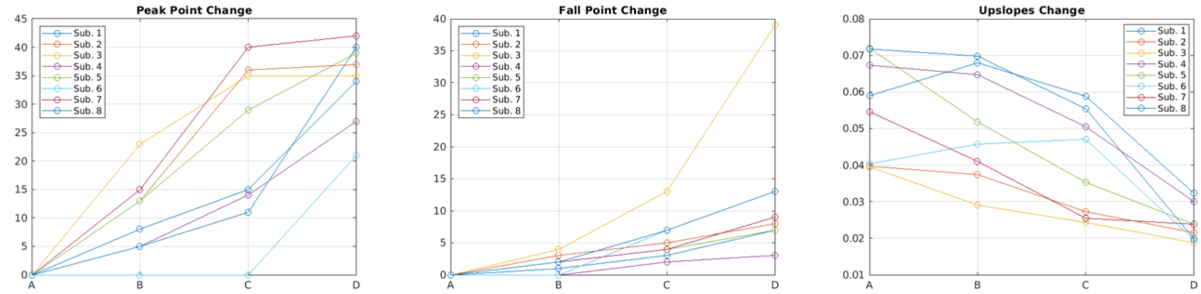

Supplementary Figure S8. Comparison of middle carpet plot subsections through various shape metrics. (Left) Change in peak point (time index, during CO_2-induced signal rise, at which the time series reached 90% of the difference between baseline signal value and mean peak signal value) relative to the bottom carpet plot section. (Center) Change in fall point (time index, during signal fall due to lowered CO_2, at which the time series lowered to 90% of the difference between baseline signal value and mean peak signal value) relative to the bottom carpet plot section. (Right) Slope of averaged time series between the breakpoint and the peak point (i.e. slope of CO_2-induced signal rise in averaged time series).

7 Transit Times for CO_2 Carpet Plots Sorted via DSC Delay Map

As an added evaluation of the similarity between CO_2 and DSC delay maps, the CO_2 carpet plots were reconstructed by sorting voxels according to the averaged DSC delay map, which was registered to each subject's native brain space. Because this resorting method prevents direct computation of the middle carpet plot section in the same way as when sorted using CO_2 delay values, the same middle carpet plot section dividing lines were used to segment the middle carpet plot section. Transit times within this section were then recomputed, with results shown in Figure S9. The resorting resulted in an apparent decrease in the clarity of the three-section carpet plot structure and had varying effects on the computed transit time. While these changes may suggest a lack of similarity in results between the DSC and CO_2 delay computation methods, it should be noted that the CO_2 transit time computation method is dependent upon subject-specific voxel sorting. Thus, since this applied sorting method is derived from an averaged map based on other subjects, inter-subject differences may account for some of the changes between carpet plot sorting methods.

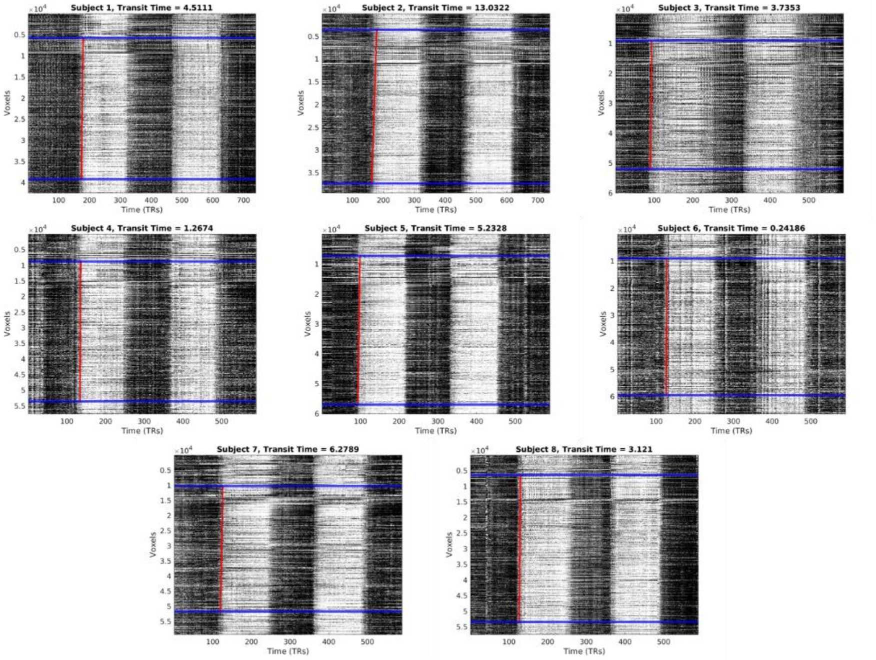

Figure S9. Carpet plots and transit times where carpet plots voxels are sorted according to the averaged DSC delay map.

8 Alternative Methods for Computing Blood Arrival Delay Times

Three additional methods were tested for computing blood arrival delay times to see if they could act as an effective replacement for the cross-correlation delay method. In the first alternative, delay times were computed as the time index of maximum signal derivative (increase) associated with the arrival of the first pass of the CO_2 bolus, similar to the method described in the main document (see Discussion) for edge detection in carpet plots. The time index of maximum signal increase was computed as the time point at which the greatest signal increase over the preceding two seconds was observed, computed after individual voxel time series were demeaned and smoothed using a 20-second Gaussian-weighted filter (MATLAB *smoothdata*). Sample results from this method for one subject are shown in Figure S10(b) and Figure S11(b), showing that the method produced noisier results than the cross-correlation method. Second, the same method was applied to compute points of maximum increase associated with both the first and second passes of the CO_2 bolus and these values were averaged to create the voxel-wise delay estimate. Sample results from this method are shown in Figure S10(c) and S11(c). This method appeared to improve slightly over the first alternative in that the overall spread of voxel delays appeared to be reduced, but the cross-correlation method still appeared to better capture vascular structure in the delay maps. Additionally, carpet plots with voxels ordered according to this second alternative are shown in Figure S12, illustrating a less clear middle carpet plot section compared with the cross-correlation method. In the third alternative, delay times were computed by estimating the break point, or point of initial signal increase caused by CO_2 arrival. Sample results from this method for one subject are shown in Figure S10(d) and S11(d), showing that the method failed to improve the wide spread of delay times observed from the cross-correlation method.

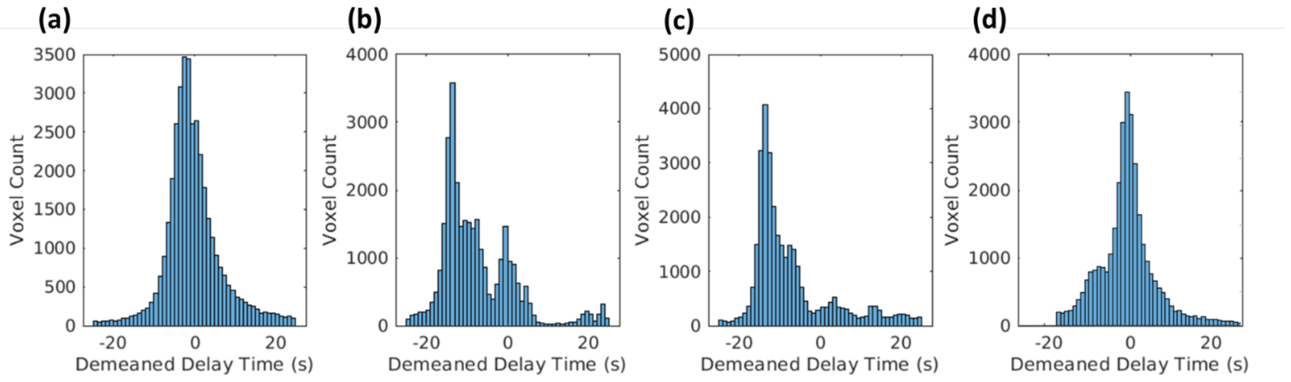

Supplementary Figure S10. Sample subject CO_2-arrival delay histograms computed using four different methods: (a) cross-correlation with the brain-averaged fMRI time series; (b) computation of voxel-specific point of maximum signal increase (as used in carpet plot slope detection); (c)

computation of voxel-specific delay as the average of point of maximum increase over both CO_2 bolus passes; (d) computation of voxel-specific estimate of breakpoint.

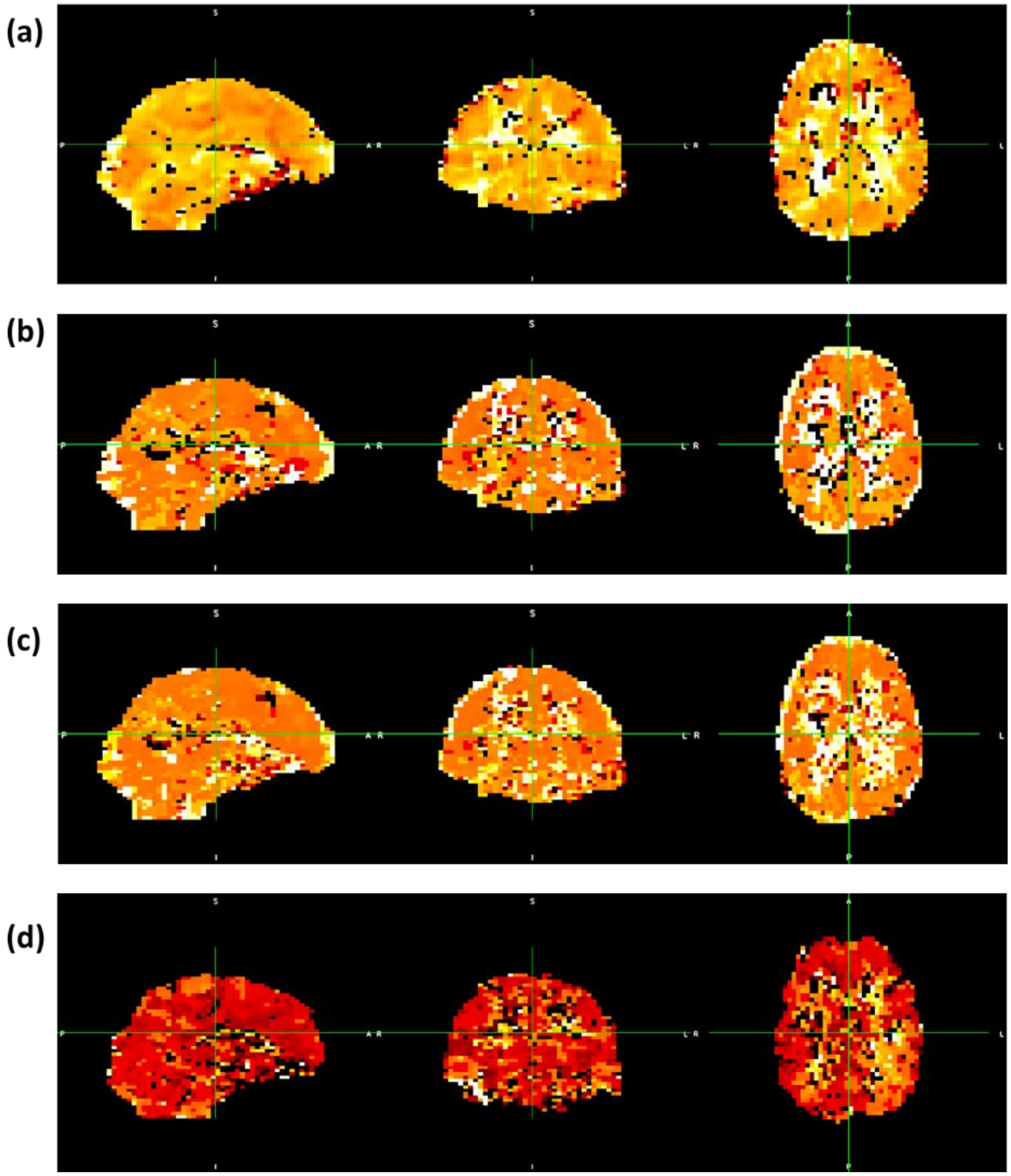

Supplementary Figure S11. Sample subject CO_2-arrival delay maps computed using four different methods: (a) cross-correlation with the brain-averaged fMRI time series; (b) computation of voxel-specific point of maximum signal increase (as used in carpet plot slope detection); (c) computation of

voxel-specific delay as the average of point of maximum increase over both CO_2 bolus passes; (d) computation of voxel-specific estimate of breakpoint.

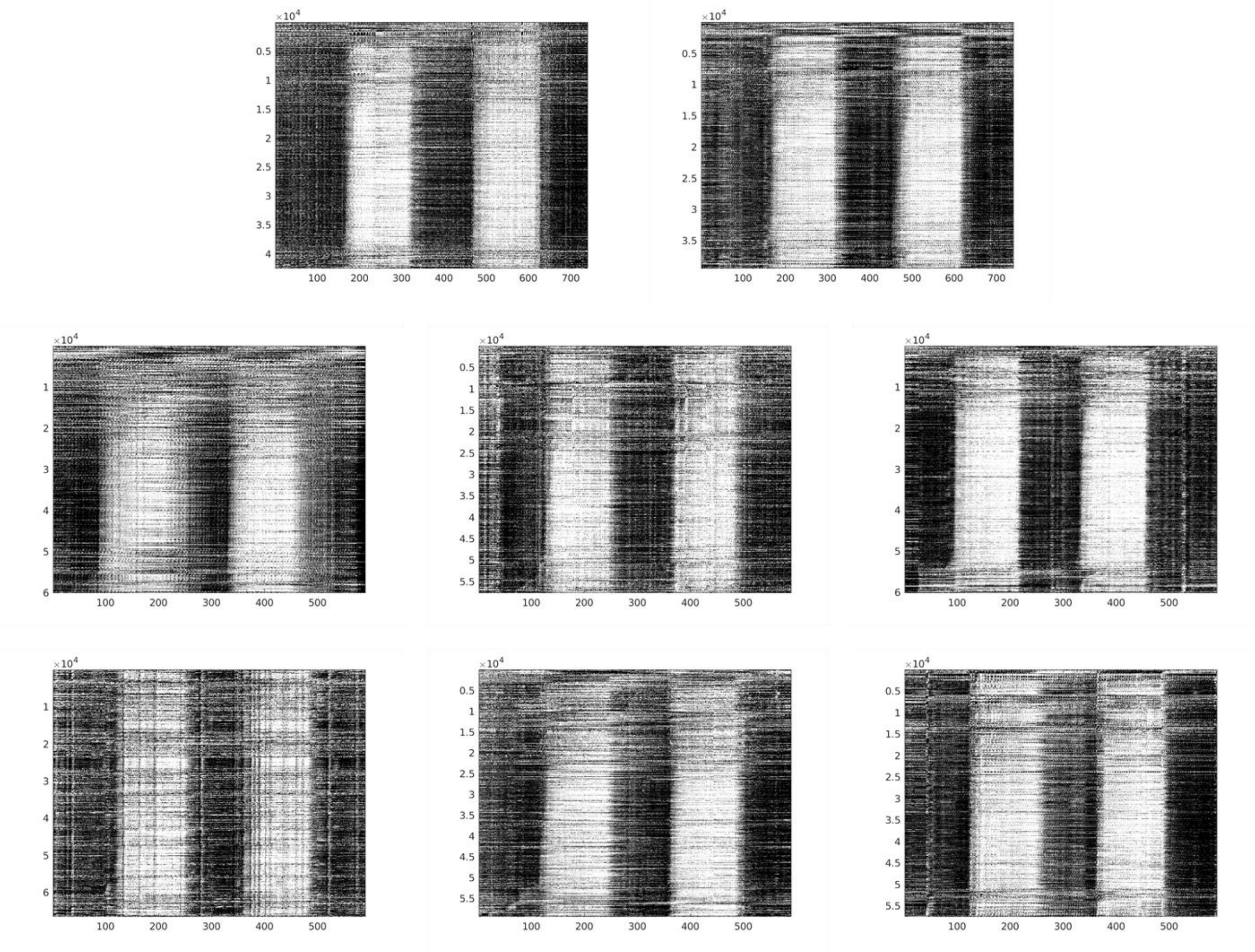

Figure S12: Carpet plots sorted by averaged maximum increase time over both CO_2 boluses.

9 Reasoning for Using Structure Element of SSIM

Figure S13 displays the results of computing the SSIM contrast and luminance element maps via comparison of the averaged CO_2 and DSC-MRI delay maps. In the SSIM metric, the contrast element measures whether the variances of the two compared windows are similar. As seen in Figure S13(a), the computed contrast map appears to be somewhat homogeneously low except for the edges (likely artificially inflated because edges will include non-brain voxels that are all the same, thus making the variances between the two maps appear more similar) and near areas of CSF, particularly in the inner ventricles. The luminance measures whether the means of the two compared windows are similar. As shown in Figure S13, the structure visible in the luminance map appears to show dark lines along paths where one or both maps have means very close to zero (especially true of the CO_2 map). Other

structures seem to just imply that the similarly of the means goes down when the window mean is further away from a zero delay. This added little new information, as the CO_2 map was already known to have a wider range than the DSC map. Unlike the structure element, both the luminance and contrast terms were highly sensitive to data normalization (by demeaning and scaling the delay maps), which added a degree of subjectivity to their interpretation. Due to these shortcomings, we chose to focus on the structure element in the main part of our study.

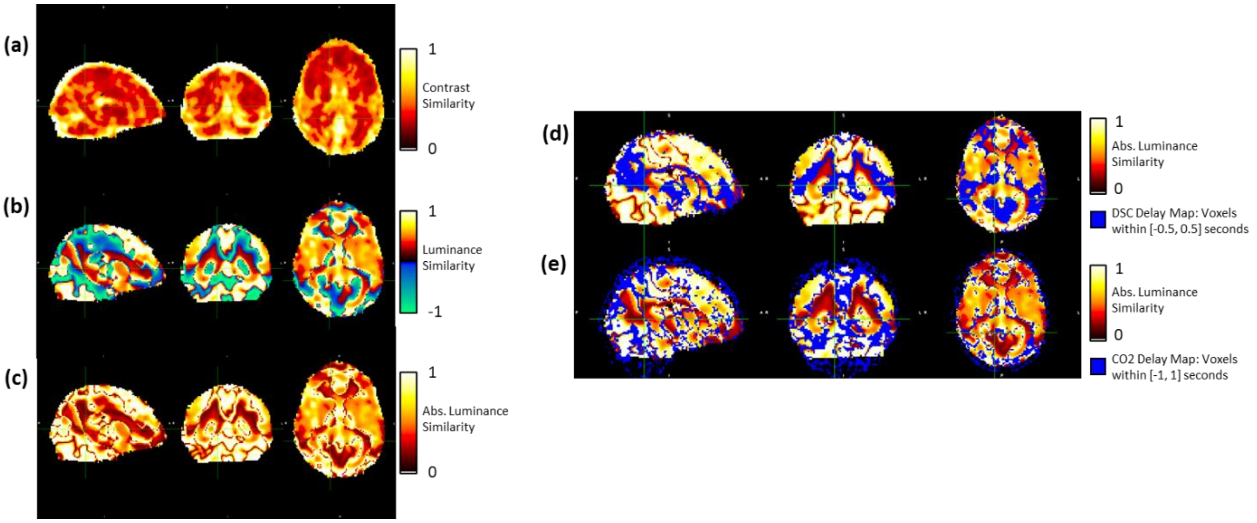

Supplementary Figure S13. Maps of contrast and luminance elements of SSIM. (a) Map of contrast element of SSIM applied to the CO_2 and DSC delay maps. The contrast measure falls within [0, 1]. (b) Map of luminance element of SSIM applied to the CO_2 and DSC delay maps. The luminance measure falls within [-1, 1]. (c) Map of absolute value of luminance element, shown to better highlight voxels with low or high magnitude. (d) Heatmap of absolute value of luminance element with overlaid map of voxels from DSC averaged delay map with delay values within [-0.5, 0.5] (near

zero). (e) Heatmap of absolute value of luminance element with overlaid map of voxels from CO_2 averaged delay map with delay values within [-1, 1] (near zero).

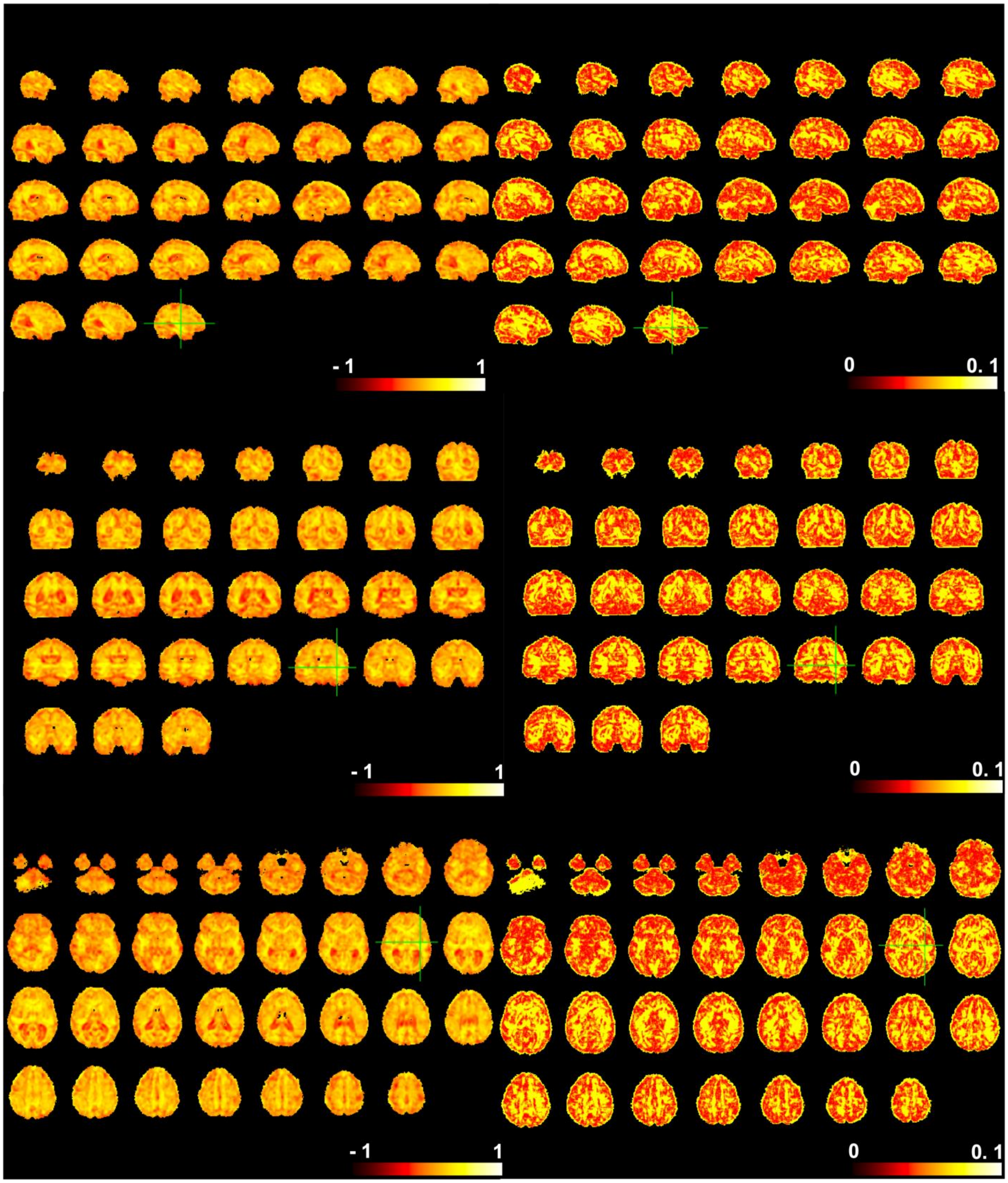

Supplementary Figure S14. Comparison of the similarity maps computed by SSIM (left) and NMI (right).

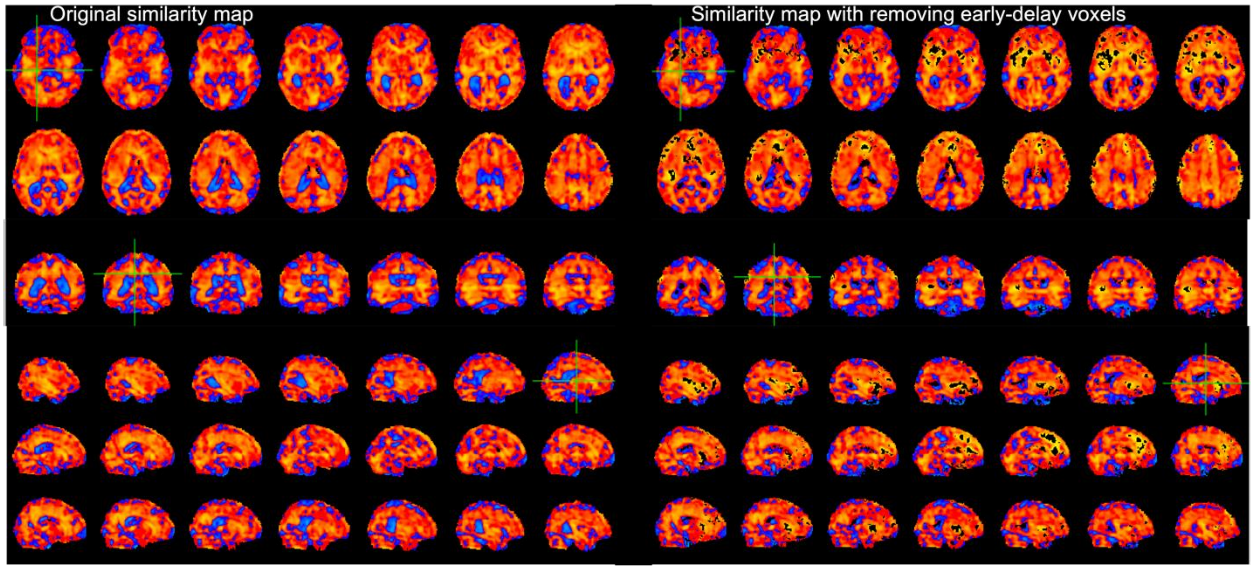

Supplementary Figure S15. The original similarity map (left) and the similarity map after removing early-delay voxels (right).

Another commonly used similarity metric, normalized mutual information (NMI), was also considered. The comparison of the spatial similarity maps derived from both metrics are shown in Figure S14. A very similar pattern of the similarity maps from both methods was observed in the majority of regions. However, in the region of the lateral ventricle, the SSIM similarity map shows low negative values, while it shows relatively high similarity values in the NMI similarity map (shown in yellow). As illustrated in Figure 4 of the main document, those voxels with low negative values are the regions showing inverted structure between the DSC and the CO_2 delay maps. Also, from Figure 6, we know that majority of those voxels are at the lower portion of the carpet plot, which means that they appeared to have earlier delay times in the CO_2 than those in the DSC, leading the inverted contrast between the CO_2 and the DSC delay maps. To further prove that these negative values are due to this inverted contrast between two delay maps, we replaced the values of those voxels with averaged delay times to remove the inverted structure between two delay maps. The resulted similarity map is shown in supplemental material Figure S15. We observed less voxels with negative values (blue region in the right side) in the lateral ventricle. Based on those, we confirmed that this is a real difference between the DSC delay map and the CO_2 delay map. This difference is successfully captured by the SSIM method, however, not by the NMI method. This capability of capturing inverted structure through negative similarity values is the main reason that we chose the SSIM approach over the NMI approach. The SSIM method is also designed to provide spatial information throughout the compared images, in contrast to a singular metric such as Pearson's correlation coefficient which yields only a single global similarity metric.

References

Fitzgerald, B., Yao, J.F., Talavage, T.M., Hocke, L.M., Frederick, B.d., and Tong, Y. (2021). Using carpet plots to analyze transit times of low frequency oscillations in resting state fMRI. *Sci Rep* 11(1)**,** 7011-7011. doi: 10.1038/s41598-021-86402-z.
